# Data-driven discovery and parameter estimation of mathematical models in biological pattern formation

**DOI:** 10.1371/journal.pcbi.1012689

**Published:** 2025-01-23

**Authors:** Hidekazu Hishinuma, Hisako Takigawa-Imamura, Takashi Miura

**Affiliations:** Department of Anatomy and Cell Biology, Graduate School of Medical Sciences, Kyushu University, Fukuoka, Fukuoka, Japan; University of Minho, PORTUGAL

## Abstract

Mathematical modeling has been utilized to explain biological pattern formation, but the selections of models and parameters have been made empirically. In the present study, we propose a data-driven approach to validate the applicability of mathematical models. Specifically, we developed methods to automatically select the appropriate mathematical models based on the patterns of interest and to estimate the model parameters. For model selection, we employed Contrastive Language-Image Pre-training (CLIP) for zero-shot feature extraction, mapping the given pattern images to latent space and specifying the appropriate model. For parameter estimation, we developed a novel technique that rapidly performs approximate Bayesian inference based on Natural Gradient Boosting (NGBoost). This method allows for parameter estimation under minimal constraints; i.e., it does not require time-series data or initial conditions and is applicable to various types of mathematical models. We tested the method with Turing patterns and demonstrated its high accuracy and correspondence to analytical features. Our strategy enables efficient validation of mathematical models using spatial patterns.

## Author summary

Biological systems show various beautiful patterns, and diverse mathematical models have been proposed to gain deep insights into the mechanisms behind the pattern formation. For example, animal coat markings show variety of attractive patterns that can be generated by Turing model. However, selecting the candidate models has been done empirically, and the experimental estimation of parameters has been costly. Recently, machine learning technologies have made remarkable progress, solving various tasks of image recognition. In this study, we utilize this machine learning technology and propose two novel data-driven methods: a method for selecting mathematical models that can generate observed patterns, and a method for estimating the parameters of the mathematical model. Using a foundation model, we convert observed patterns and mathematical model patterns into vectors in a common latent space, and the candidate mathematical models are selected according to the similarity between these vectors. Parameter estimation is performed by dimensionally reducing the vectors and inputting them into approximate Bayesian inference. We validate our method with Turing patterns, confirming that this method aligns with human visual perception and that model parameters could be estimated with high accuracy.

## Introduction

A wide variety of spatial patterns are found in living organisms. Various mathematical models have been proposed to gain insight into the mechanism of these pattern formations. These mathematical models are constructed based on knowledge and hypotheses related to the morphogenesis. It is important to verify that the mathematical models can appropriately explain the morphogenesis of interest, whereas experimental verification is difficult and incurs high costs. Thus, data-driven analysis of mathematical models by comparing target images with pattern images generated by the mathematical models is beneficial in the initial stages of verifying mathematical models. Nevertheless, there has been little research on the methods of such analysis, especially for spatial data.

Data-driven analysis of mathematical models involves two issues. One is the selection of mathematical models, identifying candidate models capable of generating patterns similar to the target images. So far, researchers have selected appropriate mathematical models empirically. This process should be more automated since different mechanisms can lead to similar patterns [1] and there are a wide variety of mathematical models to consider. Currently, selecting mathematical models is a less challenging problem than formulating the models themselves. It has been difficult to formulate mathematical models for target images flexibly using a machine learning approach. In a previous study of formulating ordinary differential equations by a machine learning approach, a large amount of time-series data is required to predict mathematical models [2]. However, only steady-state images are often available without time-series data in experiments. There are a few methods based only on steady-state images. For example, in material science, a method to formulate the appropriate model from target images was developed using the knowledge of crystallographic symmetry [3], whereas such knowledge is often not available in biology.

The second issue is parameter estimation for the mathematical model of interest. Even with the same model parameters, the model may generate slightly different images if it relies on randomly set initial values. Therefore, it is desirable to predict parameters from a few steady-state images that differ slightly depending on the initial conditions. There are several studies that can deal with parameter estimation and were tested with Turing models [1, 4]. When estimating the parameters, quantifying the uncertainty of the prediction is also useful for gaining insights into the parameters since it enables us to evaluate the robustness to perturbations of each parameter and the validity of the mathematical model. Some of the previous studies above have carried out this with the Bayesian approach [1, 3]. We propose a less constrained method that enables parameter prediction in a short time from only a few target images by completely decoupling the training and prediction stages in the approximate Bayesian estimation algorithm.

Here, we propose a comprehensive method to accomplish both the selection and parameter estimation of mathematical models for two-dimensional patterns by using machine learning. To address the problems, we embed the target or generated pattern images into vectors, which reflect the essential features of the images, by a deep learning model. The vectors enable the selection of various mathematical models and Bayesian parameter estimation considering uncertainty of the prediction by using only a few steady-state images.

## Method

### Overview

**Fig 1:**
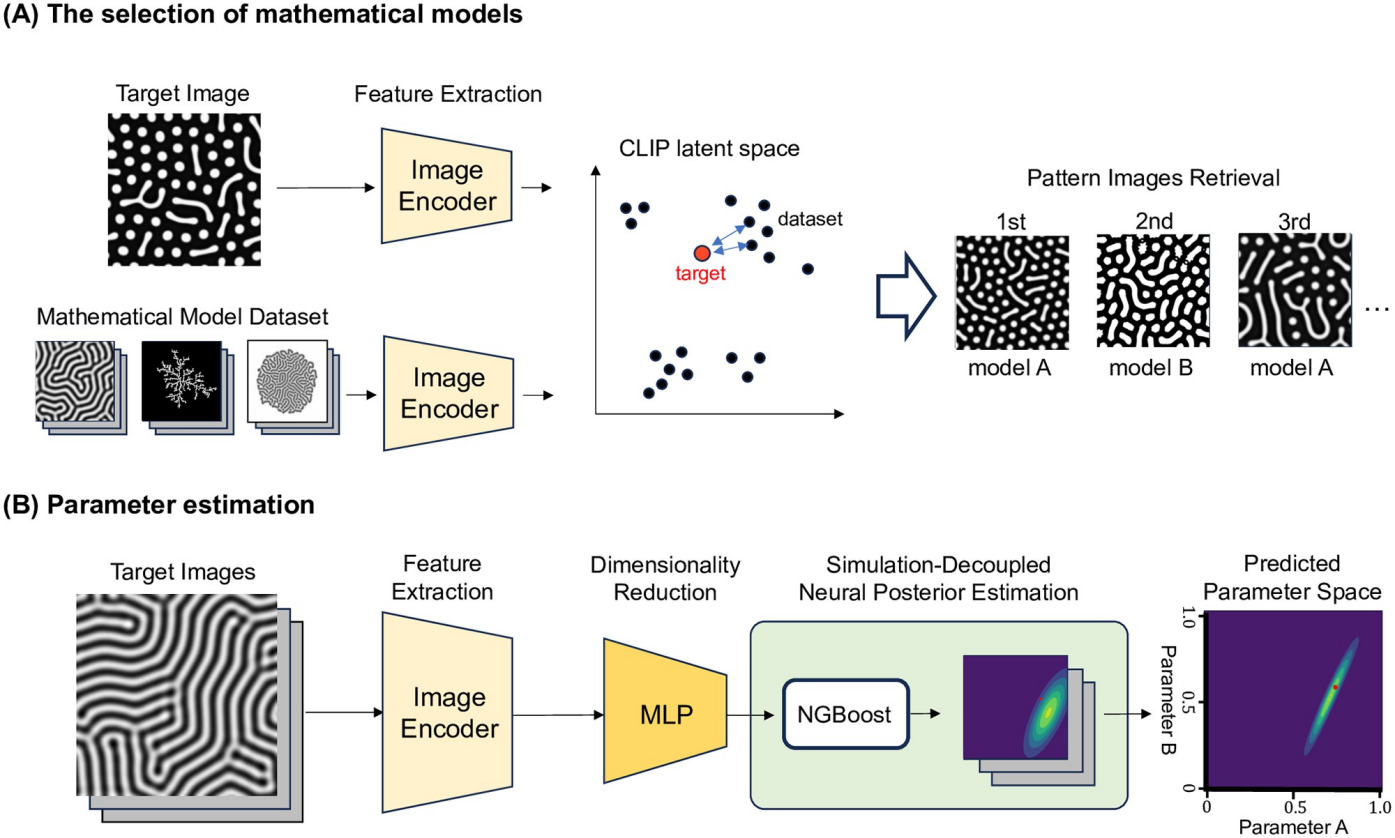
Overview. (A) The selection of mathematical models. Images from various mathematical models are embedded in Contrastive Language-Image Pre-training (CLIP) latent space. Features of a target image are extracted, and the most similar pattern images are selected. (B) Parameter estimation. Three steps, feature extraction, dimensionality reduction, and Simulation-Decoupled Neural Posterior Estimation(SD-NPE), are conducted to predict the parameters corresponding to target images generated by a single parameter set.

This section provides an overview of the computational flow of the proposed strategy. The schematic diagram is shown in Fig 1. Our strategy consists of two parts: the selection of mathematical models and parameter estimation.

In the selection of mathematical models, the first step is feature extraction. A target image was encoded into a 512-dimensional vector by the Vision Transformer (ViT) [5] image encoder, extracted from the original Contrastive Language-Image Pre-training (CLIP) model [6] and used in a zero-shot setting, meaning it was applied without any fine-tuning. Then, the similarities between the embedding vector of the target image and those of dataset including pattern images of various mathematical models were calculated. Finally, the pattern images of mathematical models with high similarity were selected.

In the parameter estimation part, the first step is feature extraction, which is common to the selection of mathematical models. One or multiple target images were embedded into CLIP latent space. In the next step, the vectors were dimensionally reduced to vectors of several dimensions. As a method for dimensionality reduction, we employed a simple multilayer perceptron (MLP) model we trained by contrastive learning. Finally, parameter estimation was performed using the dimensionally reduced vectors. For this process, we developed Simulation-Decoupled Neural Posterior Estimation(SD-NPE) that approximates Bayesian estimation using Natural Gradient Boosting(NGBoost) [7].

### Pattern image dataset

We constructed two types of datasets of pattern images from mathematical models. One dataset is a model selection dataset, and the other is a parameter estimation dataset. The model selection dataset was utilized exclusively as a database of reference patterns for similar image retrieval. Note that this dataset was not used for any machine learning training. The parameter estimation dataset was used to train the MLP for dimensionality reduction, and the NGBoost was responsible for estimating the parameters of the mathematical models.

The model selection dataset comprises pattern images of various mathematical models. In this study, we constructed the dataset based on eight mathematical models: Turing model [8], kernel-based Turing (KT) model [9], Gray-Scott model [10], Eden model [11], Diffusion-limited aggregation (DLA) model [12], phase-field model [13], Edwards-Wilkinson model [14], and L-system [15]. Each pattern image for Turing model, KT model, phase-field model, and L-system was generated from a unique set of parameters. Since the Gray-Scott model exhibits pattern changes over time, multiple time-point pattern images from a single simulation were included in the dataset. For models involving stochastic processes, such as the Eden model, DLA model, and Edwards-Wilkinson model, multiple pattern images were generated with the same parameter set. In the case of the phase-field model, only the stochastic noise applied to the initial conditions was varied. The details of the governing equations, parameter settings, and numerical simulations for each mathematical model are presented in S1 Text. The composition of the dataset is summarized in Table 1. The total number of images is 1799. The image size of all mathematical models was set to 128 × 128. We embedded the pattern images into 512-dimensional vectors by ViT and stored the vectors as the dataset instead of raw images for computational efficiency.

**Table 1:** Dataset for the selection of mathematical models.

Mathematical Model	Number of Images
Turing model	228
kernel-based Turing model	288
Gray-Scott model	486
Edwards-Wilkinson model	99
Eden model	182
Diffusion-Limited Aggregation model	300
L-system model	133
Phase Field model	83
Total	1799

For parameter estimation, we constructed a dataset for the Turing model as an illustrative example, which is independent of dataset used in model selection. The governing equations for the Turing model are defined as follows:

$$\begin{array}{c} \frac{\partial u}{\partial t}=f_{u}u-f_{v}v+qu^{2}-u^{3}+D_{u}\Delta u, \end{array}$$ (1)

$$\begin{array}{c} \frac{\partial v}{\partial t}=g_{u}u-g_{v}v+D_{v}\Delta v, \end{array}$$ (2)

where *f*_*u*_, *f*_*v*_, *g*_*u*_, and *g*_*v*_ represent the parameters of the linear terms, with *q* as the parameter of the quadratic term in the reaction component, while *D*_*u*_ and *D*_*v*_ are the diffusion coefficients. Here, *f*_*v*_ and *g*_*v*_ were selected as the target parameters for prediction. These two parameters were predicted as labels. Table 2 provides the values of the fixed parameters and the range for the parameters to be predicted. The sizes of the training dataset and validation dataset were 9,700 and 600, respectively. The details of the data generation process are presented in S1 Text.

**Table 2:** Parameter set of Turing model for parameter estimation.

Parameter	Value
*f*_*u*_	0.51
*f*_*v*_	[0.6,1.0]
*g*_*u*_	0.81
*g*_*v*_	[0.6,1.0]
*q*	0
*D*_*u*_	0.1
*D*_*v*_	1.0

### Feature extraction

In the feature extraction process, we encoded images into 512-dimensional vectors using ViT-B/32 trained with CLIP. The CLIP enables neural network models to understand images by training on a vast dataset of images paired with textual descriptions. Hereinafter, these 512-dimensional vectors will be referred to as embedding vectors, and the space of the embedding vectors will be called the CLIP latent space. We used the ViT with its model parameters fully fixed, identical to those in the original CLIP model. As a preprocessing process, we performed image blurring before embedding images to mitigate the influences of the differences in the boundary sharpness among mathematical models.

Some models generate smooth patterns (Turing, Gray-Scott, and Phase Field) while others generate binary patterns (L-system, Eden, and DLA), and the preprocessing was designed not to consider such boundary sharpness because boundary sharpness can be modulated downstream of the pattern formation mechanism in living organisms.

### Selection of mathematical models

In the selection of mathematical models, we finally displayed images with high similarity patterns to obtain insight into the selection of mathematical models. We adopted cosine similarity as the metric for similarity. The similarity between target images and pattern images in our dataset was calculated in the CLIP latent space. Since the image recognition by ViT is influenced by pattern scale, adjusting the crop size of the target image enables the search for optimal patterns. When demonstrating the selection of mathematical models with target images of living organisms, some variations of the original target images were made by cropping them in the center in increments of 5%, within the range of 50% to 100% of their length. Then, the case in which the method suggested the highest similarity image among the variations was automatically adopted. A comparison of the tendencies in similar patterns observed with different crop ratios is presented in S14 Fig.

### Dimensionality reduction

**Fig 2:**
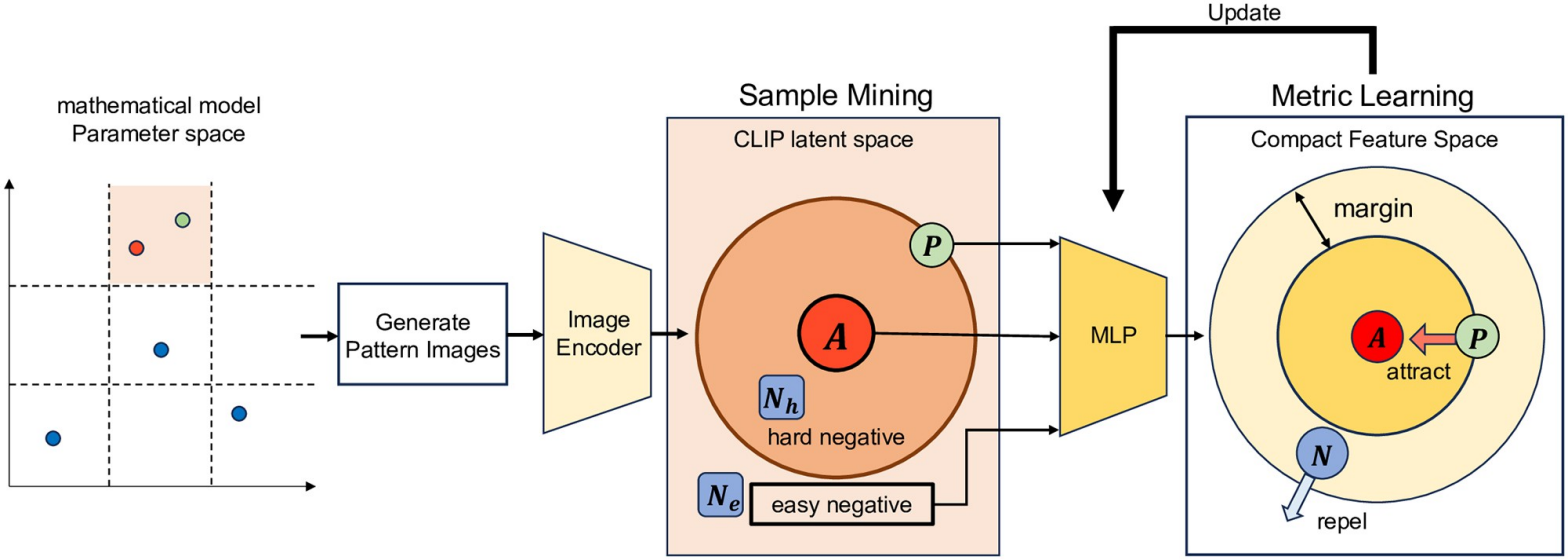
The diagram of the modified contrastive learning for dimensionality reduction. Parameter space is separated into regular grids to determine the positive and negative samples for the anchor. Only the triplets of generated images with easy negative are input to the multilayer perceptron (MLP) model, which is trained based on Triplet loss.

In the method of parameter estimation, we performed dimensionality reduction after feature extraction. We implemented an MLP model as the dimensionality reduction process. The input vectors of the model were 512-dimensional CLIP embedding vectors, and the output vectors were two-dimensional vectors, which we denote as reduced feature vectors. The number of dimensions of the output vectors should be determined depending on the number of parameters to be predicted or the complexity of the patterns. We trained the MLP model by a modified contrastive learning(Fig 2). As a loss function, we adopted Triplet Loss [16], which requires three samples for calculating the loss: an anchor sample, a positive sample, and a negative sample. In the proposed strategy, there are two modifications from standard contrastive learning. Firstly, we extended the conditional usage, which was usually applied to classification tasks, for regression tasks. For this purpose, we divided the parameter space into grids. Then, pattern images located in the same grid as the anchor were regarded as positive samples, while those outside the grid were regarded as negative samples. The size of the grids was a hyperparameter. Secondly, taking into account the fact that different parameters could correspond to the similar pattern, we established conditions for excluding triplets from the calculation of the loss. Specifically, the condition is that the similarity of the negative sample is higher than that of the positive sample in the CLIP latent space. The above mining method was implemented with another method, multi-similarity mining [17].

The dataset for contrastive learning was composed of 512-dimensional embedding vectors and the grid numbers of parameters as labels. The details of the MLP model are summarized in Table 3. The settings of the batch normalization and dropout layer were default, and we did not test the other conditions. The settings of contrastive learning are also shown in Table 4. When training the model, we used early stopping to avoid overfitting. Early stopping was applied using the default settings in PyTorch Metric Learning. Specifically, instead of monitoring training loss, the Mean Average Precision at *r* (MAP@*k*) score, an evaluation metric for deep metric learning, was tracked during training. The training was stopped if the maximum MAP@*k* score did not improve for 20 epochs. The MAP@*k* score was calculated based on the *k*-nearest neighbors of each data point in the learned latent space, and *k* represents the total number of samples with the same label. The method for calculating MAP@*k* follows S4 Text. S16 Fig shows the progress of the training loss and MAP@*k* score over the course of training. Therefore, the number of epochs was at most 30, though the maximum number of epochs was set to 100.

**Table 3:** The configuration of the architecture for dimensionality reduction.

Layer	# nodes	Activation function	Batch normalization	Dropout
Input layer	512	-	-	-
Hidden layer 1	256	ReLU	+	0.2
Hidden layer 2	128	ReLU	+	0.2
Hidden layer 3	128	ReLU	-	-
Hidden layer 4	64	ReLU	-	0.2
Hidden layer 5	64	ReLU	-	-
Hidden layer 6	32	ReLU	-	-
Hidden layer 7	32	ReLU	-	-
Hidden layer 8	16	ReLU	-	-
Output layer	2	-	-	-

**Table 4:** Training Conditions for Contrastive Learning.

Training Parameter	Value
Epoch	≤ 30
Batch Size	32
Learning Rate	0.001
Weight Decay	0.001
Margin of Triplet Loss	0.1
Epsilon of Multi-Similarity Miner	0.2
Number of Grids	10 × 10

### Simulation-decoupled neural posterior estimation

SD-NPE is the final and central process of the proposed method of parameter estimation. In this process, the module takes an arbitrary number of reduced feature vectors, which is the output of the dimensionality reduction model, and outputs the numerical result of approximate Bayesian inference of parameters. The calculation consists of two steps. The first step is the computation of NGBoost for each input vector, and the second step is a comprehensive calculation that integrates the outputs from the first step. This comprehension allowed for the prediction of parameters from multiple target images, though NGBoost just takes the fixed-length vectors as input.

In preparation for this method, We trained NGBoost (Fig 1) to predict the parameters with uncertainty from one input vector. The dataset for training NGBoost was composed of reduced feature vectors of Turing model and their corresponding parameters as the labels. We specified a multivariate normal distribution as the parametric probability distribution that NGBoost should predict. The other details are summarized in Table 5.

**Table 5:** Training Conditions for NGBoost.

Training Parameter	Value
Criterion of Decision Trees	Friedman MSE
Max Depth of Decision Trees	3
Number of Estimators	150
Learning Rate	0.087
Lattice Size of Parameter Space	0.005
Number of Lattices	100 × 100

In the calculation of the second step, we need the prior distribution of the parameter space. The pixel-wise average of the distributions of all samples predicted by the NGBoost was calculated. It was regarded as the approximation of the prior distribution of the parameter space for the NGBoost model under Monte Carlo integration.

Finally, in the second step, Bayesian inference of parameters was performed under mean-field approximation by using the approximate prior distribution and the predictions from the first step. The derivation of this method is described in S2 Text.

## Result

### Selection of mathematical models by CLIP

**Fig 3:**
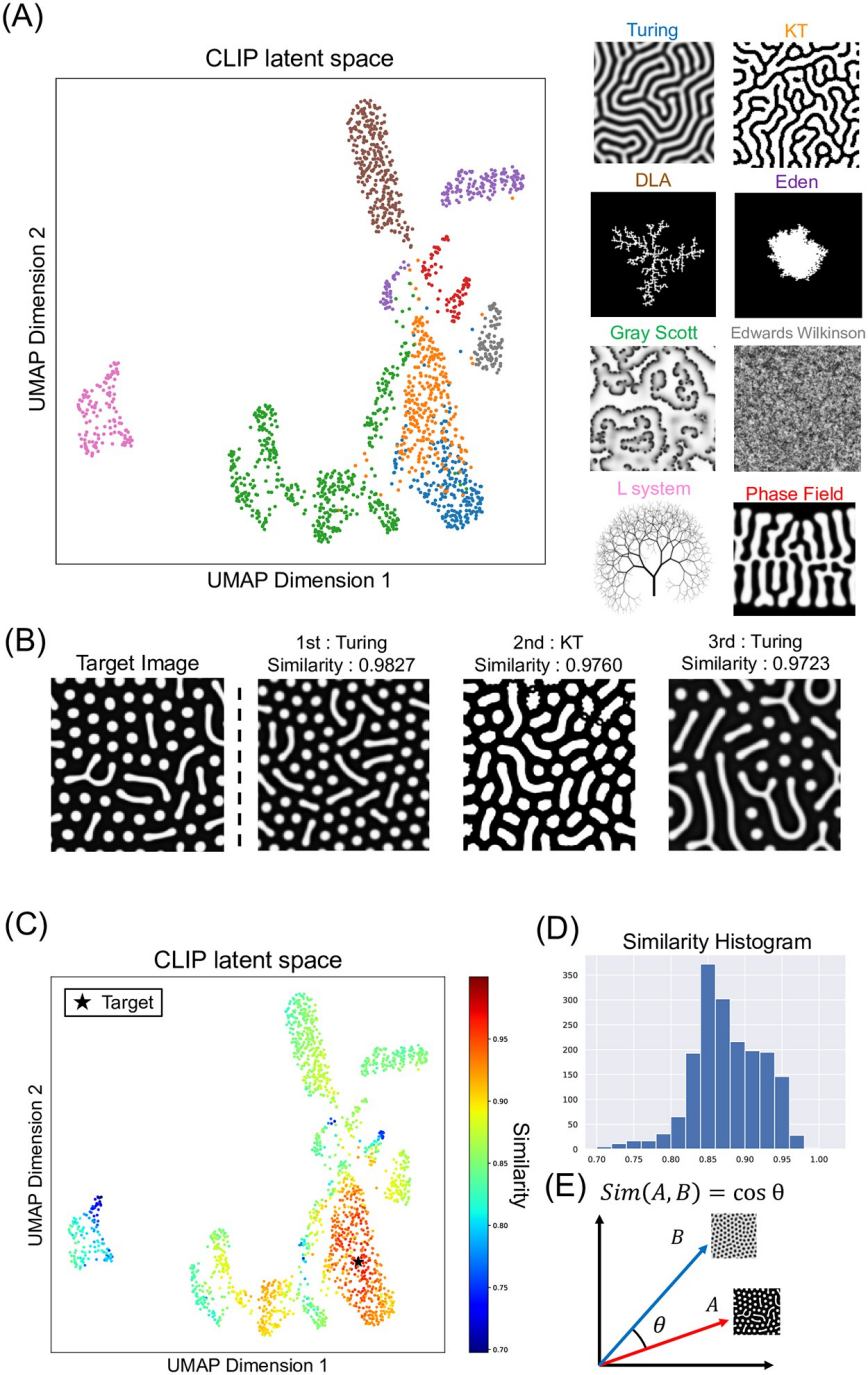
Model selection by CLIP. (A) Mapping of mathematical models in Contrastive Language-Image Pre-training (CLIP) latent space. The figure shows the results of visualizing CLIP’s 512-dimensional latent space, reduced to two dimensions using Uniform Manifold Approximation and Projection (UMAP). The horizontal and vertical axes represent the first and second components of the two-dimensional vectors obtained from UMAP, respectively. (B) The target image and the top three images with the highest similarity. (C) Representation of cosine similarity to the target Turing image. The figure shows the same scatter plot as in (A), with colors indicating the similarity to the target example. Star shows the position of the target image. The horizontal and vertical axes represent the first and second components of the two-dimensional vectors obtained from UMAP, respectively. (D) The histogram of cosine similarity scores of all datasets to the target image. (E) The definition of cosine similarity.

Our strategy can exploit CLIP’s latent space to search for similar pattern images, which helps to screen various mathematical models. For clarity, we highlight that the model parameters of ViT were kept identical to those in the original CLIP model, with no additional fine-tuning performed in this study. As a preliminary experiment, we verified that CLIP, as a multimodal foundation model, sufficiently recognizes not only general-purpose images but also the geometric features inherent in datasets from mathematical models, even in a zero-shot setting (S5 Text and S8 and S9 Figs). Then, we examined the similarity relationships between mathematical models within CLIP’s latent space. Dimensionality reduction by Uniform Manifold Approximation and Projection (UMAP) was used to represent embedding vectors in a two-dimensional scatter plot (Fig 3A). We applied clustering algorithms, and the mathematical models were separated into some groups, which reflect the textures like graph, lattice, and partial differential equations (PDE) (Fig 3A and S10(A) Fig). The graph-like model, L-system, is in the left of the plot. The lattice-like models, such as the DLA model and Eden model, are at the top right, and the PDE models are at the bottom right. Turing model, KT model, and some samples of Gray-Scott model were assigned to the same cluster.

This system can be used to find images from the dataset that are similar to a particular given target image. We tested the method using a pattern image of Turing model as the target. To find images similar to the target image, cosine similarity was employed as a metric for assessing similarity since the image encoder, ViT was trained by CLIP with cosine similarity in the previous study(Fig 3E). The top three images with the highest cosine similarity were retrieved (Fig 3B). The image with the highest similarity was generated from the same Turing model and extremely similar to the target image. The second image was generated from KT model, suggesting that this method can retrieve similar images from multiple mathematical models. Images with high similarity ranked fourth and below are shown in S12 Fig. The similarity to the target was overlaid on the Fig 3A to examine the trend of similarity across all datasets (Fig 3C). It shows that most of the simulation data with high similarity belong to Turing model or KT model (Fig 3A and 3B and S10(B) Fig).

**Fig 4:**
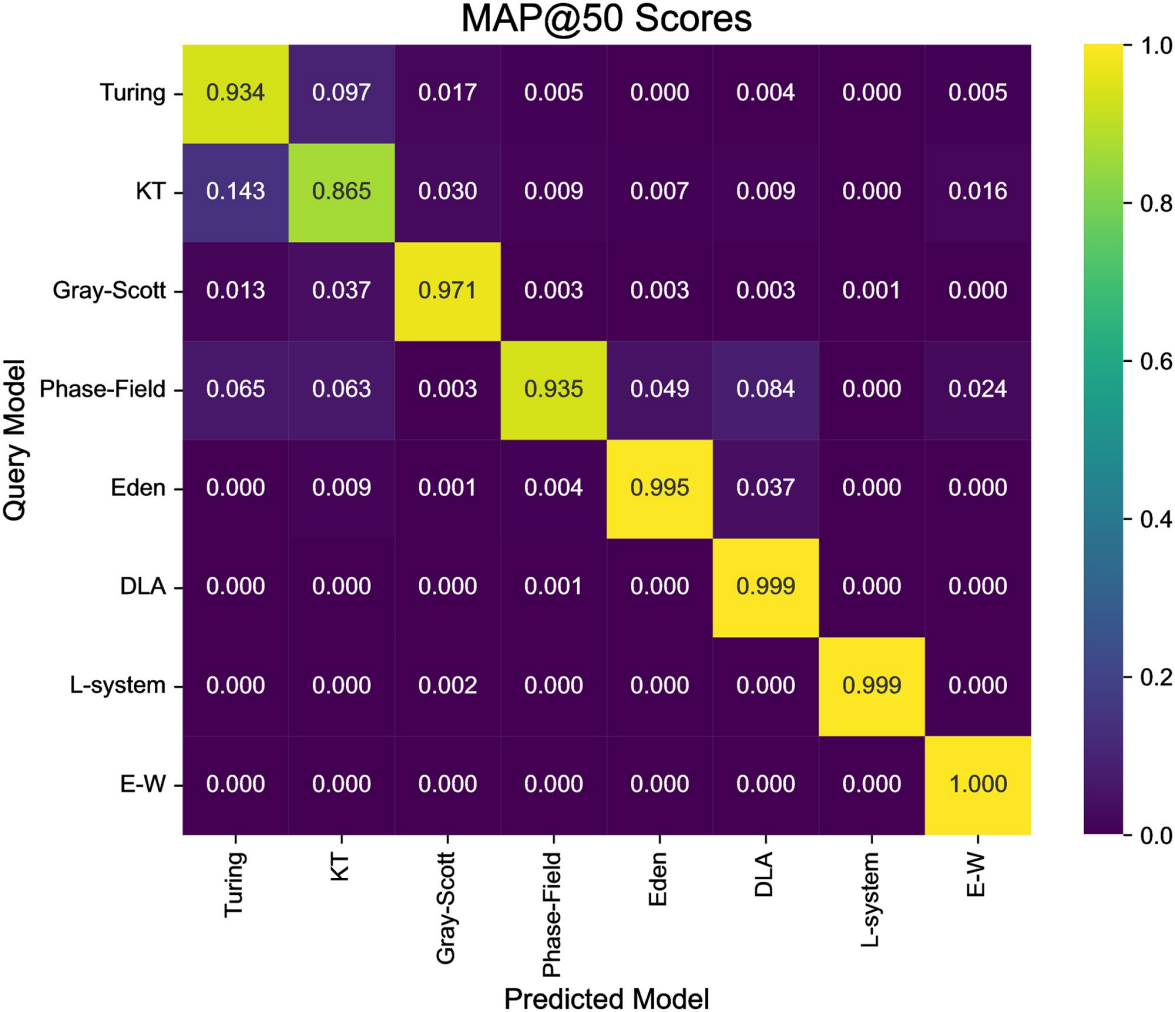
Heatmap of MAP scores between patterns of mathematical models. The heatmap displays the average Mean Average Precision (MAP) score for patterns that rank within the top 50 in similarity when a pattern image of a mathematical model is used as input. The vertical axis represents the type of mathematical model used as input, while the horizontal axis represents the type considered as the true label for MAP calculation. The range of possible MAP scores is [0, 1].

When searching for similar patterns to a target image of a mathematical model, the same model usually ranked higher. The tendencies in the recommendation rankings for each mathematical model were quantitatively assessed. Note that the purpose of the model selection is not to identify identical mathematical models but rather to discover any models capable of generating similar patterns to the target images. We applied Mean Average Precision at *k* (MAP@*k*), a metric commonly employed in recommendation systems. MAP@*k* calculation only includes the top *k* similar pattern images. Here, *k* was set to 50 to mitigate the impact of data imbalance. In the MAP@50 score matrix (Fig 4), the score in the *i*-th row and *j*-th column represents the degree to which the pattern images generated by the *j*-th mathematical model are concentrated at high ranks when the *i*-th mathematical model is used as the target image. The details of the MAP@*k* calculation were described in S4 Text. In the search for images similar to those generated by the Turing model, images from the KT model were frequently found among the higher ranks, and the reverse was also observed. These results are consistent with the fact that KT model can theoretically generate patterns identical to the Turing model, though KT model differs in texture due to the simulation techniques compared to conventional PDE models.

Next, we evaluated the response of our method to target pattern images generated by parameter sets outside the range specified when creating the model selection dataset (S1 Text) or by mathematical models not included in the dataset (S11 Fig). While dissimilar pattern images were sometimes selected when the pattern scale was too small relative to the image size, in most cases, our method was able to find similar patterns within the dataset.

**Fig 5:**
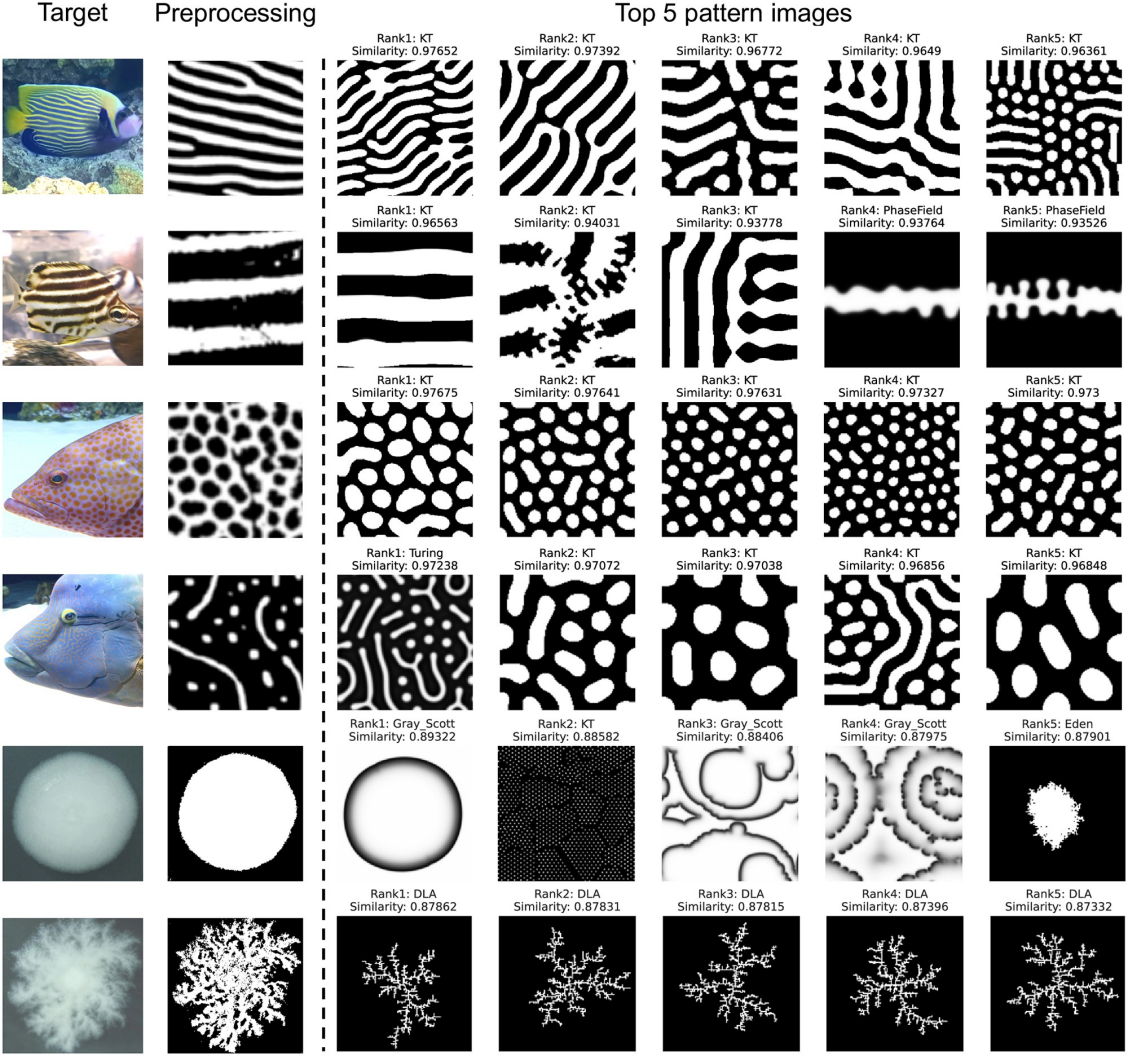
Images of living organisms and high similarity patterns selected by CLIP. Original images of living organisms (The leftmost column), target images after preprocessing (The second column from the left), and pattern images of top 5 similarities in the latent space of Contrastive Language-Image Pre-training (CLIP)(The third column from the left onwards). The target living organisms are emperor angelfish (*Pomacanthus imperator*), stripey (*Microcanthus strigatus*), areolate grouper (*Epinephelus areolatus*), humphead wrasse (*Cheilinus undulatus*), and mutants of *Bacillus mycoides Flügge* [18] from top to bottom.

We finally tested the method using images of actual living organisms. Six examples of application to living organisms are shown in Fig 5. For the fish skin pattern with stripes, spots, or both, similar patterns generated from Turing model and KT model were selected. For images of bacterial colonies [18], DLA model and Eden model were selected, though the Gray-Scott model was also included due to its shape similarity. Using humphead wrasse (*Cheilinus undulatus*) as an example, similar pattern images and detailed statistics of similarity are shown in S13 Fig. Based on human judgment, the image with the highest similarity particularly captured the characteristics of the skin pattern. Thus, it was demonstrated that this method can be utilized to identify a mathematical model that represents the target image.

### Parameter estimation

#### Validation of simulation-decoupled neural posterior estimation

First, we compared the result of SD-NPE with that of analytical Bayesian inference in linear regression models (S1 and S6 Figs) and verified that SD-NPE could produce correct outcomes. This test comprised two sets of conditions: simple and redundant. Under the simple condition, our method approximated Bayesian inference with very high accuracy(S2 Text and S1 Fig). The redundant condition was assumed for cases where parameters could not be uniquely determined. The precision was not as high as in the simple condition; however, the approximated prior distributions also resembled analytical solutions (S6 Fig). Details of this verification are described in S2 Text.

**Fig 6:**
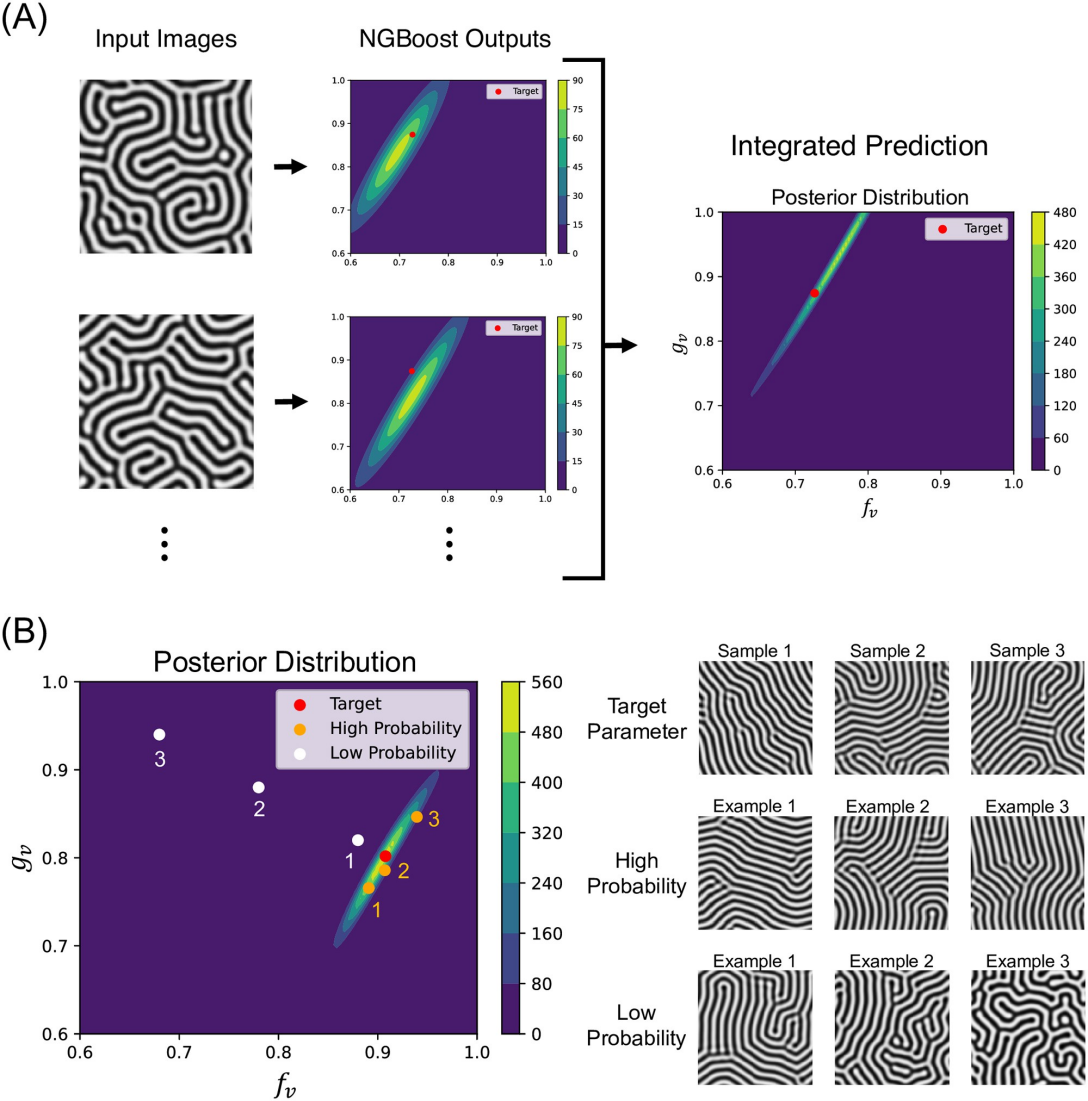
Parameter estimation of the Turing model. (A) The process of Simulation-Decoupled Neural Posterior Estimation(SD-NPE). For prediction, each data sample is input into Natural Gradient Boosting (NGBoost) individually, which outputs a posterior distribution of parameters for that sample. By integrating these posterior distributions with precomputed approximate prior distributions, we can approximate the posterior distribution of parameters across all samples. (B) An example of the estimation of parameters *f*_*v*_ and *g*_*v*_ of the Turing model. Left: the approximated posterior distribution and the plots of points of target parameters and parameters with high and low probability. Right: Each line shows three examples of the Turing model images corresponding to the target, high, and low probability parameters, respectively.

Next, we applied our method to an actual mathematical model, Turing model. The target images for parameter estimation were generated by the Turing model. A rough estimation by NGBoost for each target image was integrated into a fine estimation, which represented the approximated posterior distribution given all samples of the target images(Fig 6A). An example of the prediction is presented in Fig 6B. In this example, the prediction was computed from 20 samples of target images with the same parameters *f*_*v*_: 0.9078, *g*_*v*_: 0.8018. It can be seen that the true parameters existed on the high probability region of the predicted distribution(Fig 6B). There were few differences among the target images and the images with high probability according to the prediction, and the posterior distribution clearly excluded parameters such that the patterns were different from the target images. Interestingly, some parameters corresponding to apparently similar images were also excluded from the distribution (low probability example 1 in Fig 6B), suggesting that the method could perform a more precise parameter estimation taking into account features that humans do not intuitively understand. Here, the parameters of high probability images were probabilistically sampled from the predicted distribution and those of low probability images were selected manually. The patterns corresponding to each point on the entire parameter space are shown in S15 Fig.

**Fig 7:**
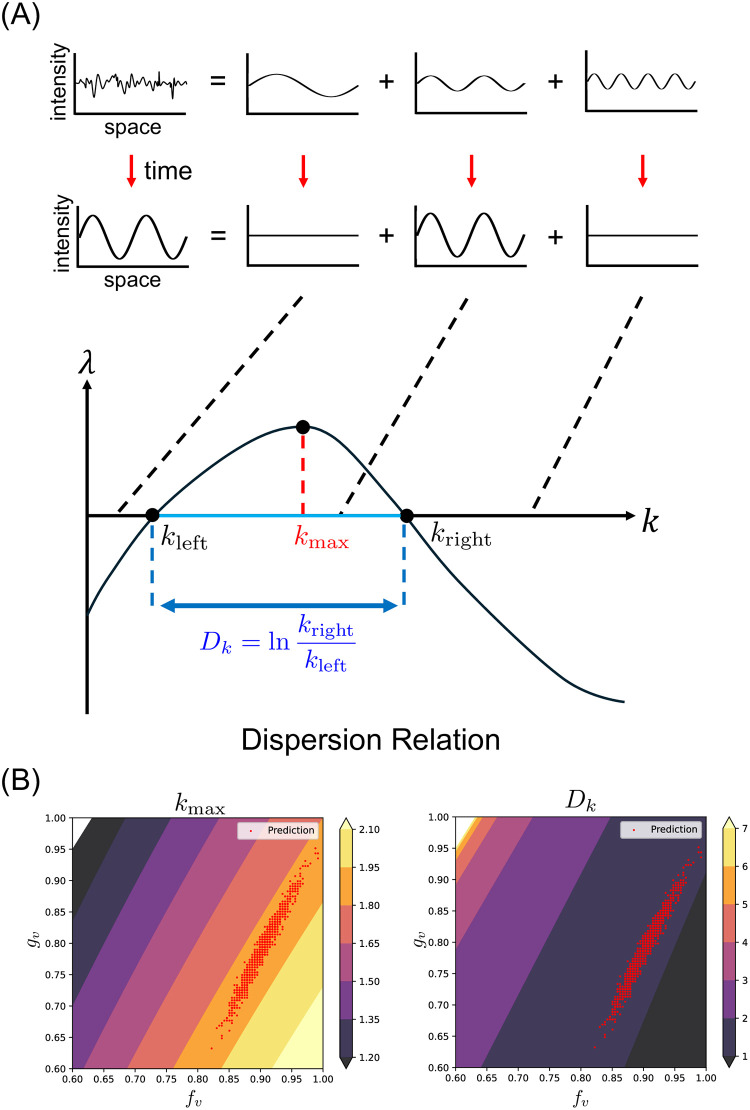
Turing mechanism and the analytical features. (A) Relationship between analytical solution and predicted probability distribution. Probabilistic samplings of (*f*_*v*_, *g*_*v*_) (red) are mapped on the contour plot of analytically-obtained pattern characteristics (*k*_max_ and *D*_*k*_. (B) Linear stability analysis of Turing pattern. The dispersion relation shows the relationship between the wavenumber *k* and the growth rate λ of its amplitude. *k*_max_ is the *k* when λ is at its maximum. *D*_*k*_ is calculated from *k*_right_ and *k*_left_, which are defined as the bounds on the range of *k* when λ is positive [20].

Then, we examined the interpretation of the predicted linear-like distribution compared with the analytical characteristics of Turing model. In Turing model, patterns grow at a specific spatial scale such as the width of stripes or the spacing between spots. Here, we refer to this spatial scale as wavenumber. The dispersion relation shows the features of Turing patterns, focusing on the growth of wavenumber(Fig 7A). We denote the most prominently growing wavenumber as *k*_max_ and the range of wavenumber that has positive λ as *D*_*k*_.

*k*_max_ reflects the main spatial scale of the pattern [19] and *D*_*k*_, which was designed in a previous study [20], reflects whether it is close to a labyrinthine pattern rather than a straight-striped pattern. *k*_max_ and *D*_*k*_ are defined as follows:

$$\begin{array}{c} k_{\text{max}}=\sqrt{\frac{D_{u}D_{v}\left(f_{u}-g_{v}\right)+\left(D_{u}+D_{v}\right)\sqrt{-D_{u}D_{v}f_{v}g_{u}}}{D_{u}D_{v}\left(D_{v}-D_{u}\right)}}, \end{array}$$ (3)

$$\begin{array}{c} D_{k}=\text{ln}\;\frac{A+\sqrt{B}}{A-\sqrt{B}}, \end{array}$$ (4)

where

$$\begin{array}{c} A=D_{v}f_{u}+D_{u}g_{v}, \end{array}$$ (5)

$$\begin{array}{c} B=D_{v}^{2}f_{u}^{2}+2D_{u}D_{v}\left(2f_{v}g_{u}-f_{u}g_{v}\right)+D_{u}^{2}g_{v}^{2}. \end{array}$$ (6)

Fig 7B shows the relationship between the prediction and the analytical features (i.e. *k*_max_ and *D*_*k*_). The direction of the predicted distribution was closely aligned with each analytical feature, especially *k*_max_. When substituting the constants, except for *f*_*v*_ and *g*_*v*_, into Eq 3, we obtain the following expression:

$$\begin{array}{c} k_{\text{max}}=3.3\sqrt{0.1\left(0.51-g_{v}\right)+0.31\sqrt{f_{v}}}. \end{array}$$ (7)

Within the range of possible values for *f*_*v*_ and *g*_*v*_, the contour lines of *k*_max_ exhibit a monotonic shape. The fact that the probability distribution of the parameters estimated by our method takes a linear form may correspond to this tendency of *k*_max_. Unfortunately, it was impossible to directly explain what features ViT captured from pattern images due to the ‘black box’ nature of neural network models.

#### Differences between dimensionality reduction by a neural network and that by UMAP

Although ViT can extract the features of pattern images, the latent space is high-dimensional, and the space should be dimensionally reduced for parameter estimation (S3 Text). In order to minimize the dimensions of the embedding vectors, we trained an MLP model with modified contrastive learning.

**Fig 8:**
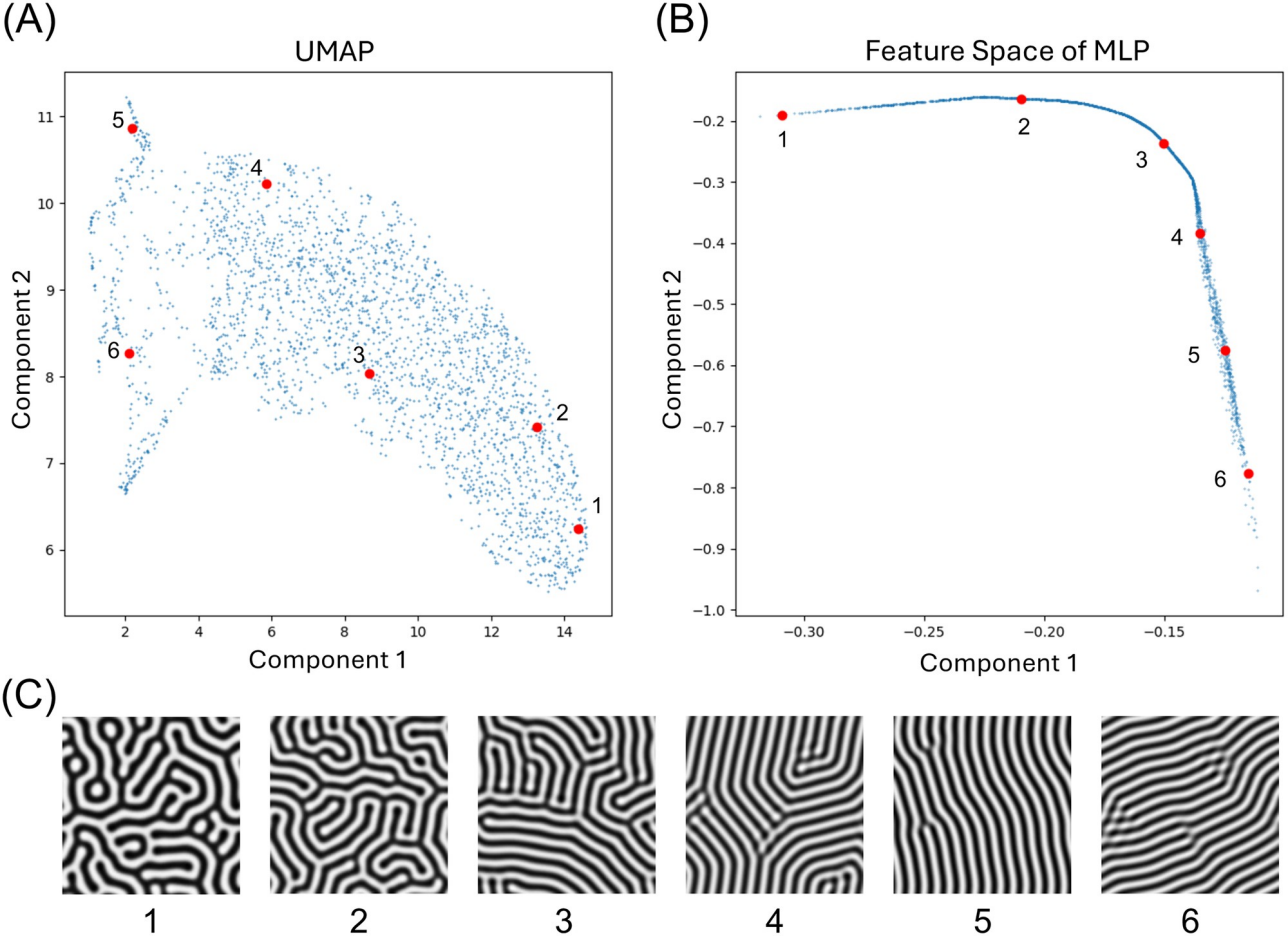
Comparison between UMAP and the MLP trained with contrastive learning. (A) Dimensional reduction by Uniform Manifold Approximation and Projection (UMAP). The horizontal and vertical axes represent the first and second components of the two-dimensional vectors obtained from UMAP, respectively. (B) Dimensional reduction by multilayer perceptron (MLP). The horizontal and vertical axes represent the first and second components of the two-dimensional output vectors of MLP, respectively. (C) The Turing pattern images of positions 1–6 in the feature space of UMAP (A) and that of MLP (B).

We compared the tendencies of the space after dimensionality reduction by the MLP model and by UMAP (Fig 8B and 8C). While the UMAP result was dispersed, the MLP model result showed a single curve. Both UMAP and the MLP model reflected some characteristics of the Turing pattern (Fig 8C). We sampled six images from different locations in the feature space reduced by the MLP model (Fig 8B and 8C) and confirmed that pattern images were mapped with tendency of whether the lines were aligned in the same direction. Similarly, the feature space of UMAP also preserved these tendencies to some extent (Fig 8A).

#### Influences of dimensionality reduction on the predictions

**Fig 9:**
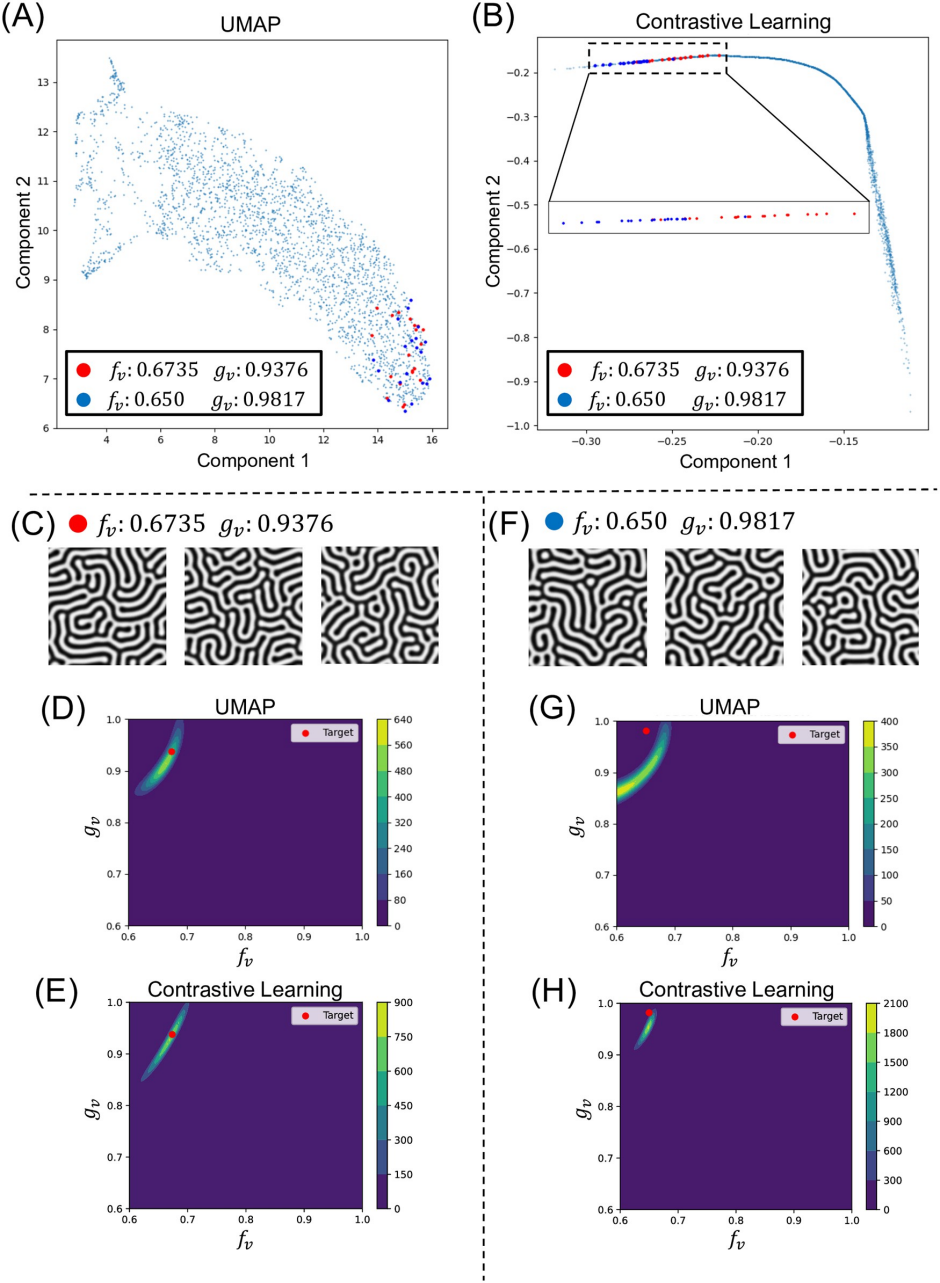
Comparison of dimensional reduction of similar patterns. (A) Distribution of patterns in Uniform Manifold Approximation and Projection (UMAP) feature space. (B) Distribution of patterns in contrastive learning feature space. (C-E) Examples of Turing patterns and predicted posterior distributions for (*f*_*v*_ = 0.6735, *g*_*v*_ = 0.9376) (Red points in (A-B)): (C) pattern examples, (D) UMAP-based dimensionality reduction, (E) contrastive learning-based dimensionality reduction. (F-H) Examples of Turing patterns and predicted posterior distributions for (*f*_*v*_ = 0.650 and *g*_*v*_ = 0.9817) (Blue points in (A-B)): (F) pattern examples, (G) UMAP-based dimensionality reduction, (H) contrastive learning-based dimensionality reduction.

To qualitatively verify the effect of contrastive learning approach for dimensionality reduction, we examined the distribution of two types of pattern images after each dimensionality reduction(Fig 9A and 9B). These pattern images were derived from either of two types of parameters which can generate very similar labyrinth-like patterns(Fig 9C and 9F). In the space composed by the outputs of our MLP model, these two types of distributions are roughly separated allowing for overlapping of some samples, whereas the distributions on that of UMAP were mixed. Our method was able to distinguish the two types of parameters and predicted their posterior distributions more clearly (Fig 9D, 9E, 9G and 9H).

Secondly, we compared the accuracy of parameter estimation among three methods(i.e., modified contrastive learning, UMAP, and without dimensionality reduction). We defined the generalization error as a measure of the accuracy according to the following:

$$\begin{array}{c} \text{Error}=-\frac{1}{N}\sum \text{log}\;p\left(w^{*}|X\right). \end{array}$$ (8)

**Fig 10:**
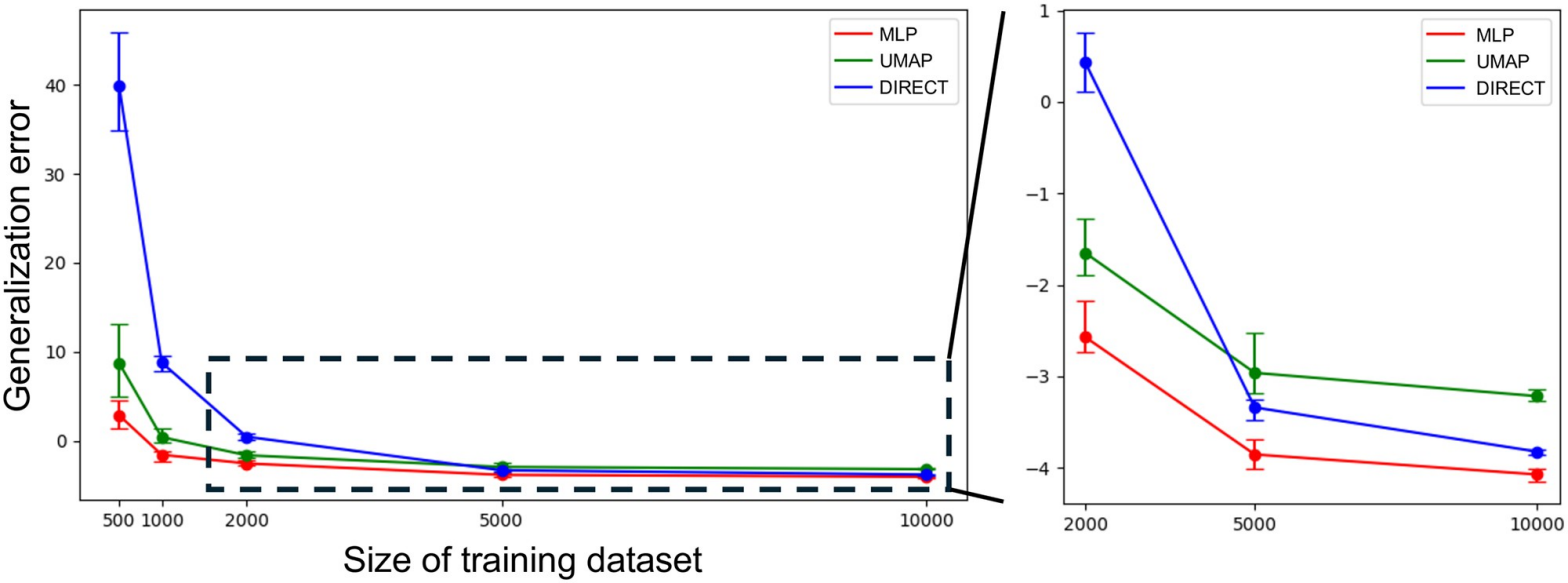
Comparison of the prediction errors with MLP model, UMAP, and without dimensionality reduction. The horizontal axis represents the number of samples used for training. The vertical axis represents the value of the generalization error. Error bars indicate the maximum and minimum values among the five training sessions. The multilayer perceptron (MLP) model trained with contrastive learning (red) was more efficient than both Uniform Manifold Approximation and Projection (UMAP)(green) and the case without dimensionality reduction (blue), even when the sample size was large (indicated by the dashed box).

The error was calculated based on the predicted probability density at the points of the correct parameters. Here, *X* represents multiple images generated from the same parameters, w^*^ is the correct parameter, and *p*(*w* ∣ *x*) is the predicted posterior distribution by SD-NPE. The test data consists of *N* groups of correct parameters and corresponding multiple images. In our experiments, *N* was 350. We tested the three methods changing the size of training dataset. Taking into account the variability in model performance due to the random seed used during training, we trained the models five times for each condition and collected statistics of the generation error. In all cases of the dataset size, our MLP model exhibited the smallest generalization error, indicating its high accuracy (Fig 10). When the data size was small, such as less than 1000, the effect of dimensionality reduction to the generalization error was significant. Interestingly, the case of UMAP underperformed compared to the case without dimensionality reduction when there were sufficient samples, whereas contrastive learning method consistently had the lowest error(Fig 10). From the above results, it was found that the dimensionality reduction using our modified contrastive learning is the most effective for parameter estimation. In addition, as the data size increases, the variance of the generalization error decreases(Fig 10). This means that the accuracy of the models trained with larger dataset are less affected by the random seed. The examples of the prediction by the models trained with datasets whose size were 2000 or 10000 were shown in S17 Fig. As can be seen from this result, a sufficient number of samples leads to more stable learning even though our method is effective with a small number of samples.

## Discussion

In this study, we developed general methods for identifying mathematical models that exhibit patterns of interest, and for performing Bayesian estimation of the parameters of a given mathematical model. In the machine learning field, the general feature extraction method from pattern images and the cost optimization of approximate Bayesian estimation are two major challenges. We enabled feature extraction by mapping patterns from various mathematical models to the same latent space by utilizing the foundation model ViT without fine-tuning. We demonstrated that the embedding by the neural network appropriately functions as feature extraction for images. Also, we developed an approximate Bayesian estimation of parameters by interpreting the regression model NGBoost, which outputs prediction and uncertainty, within the context of Bayesian statistics.

Mathematical models can generate synthetic data depending on the model parameters, but the parameters usually cannot be predicted explicitly from the data because their likelihood is typically intractable. Such scenarios have led to the development of Bayesian inference approaches known as likelihood-free inference or Simulation-Based Inference, which have been actively researched in recent years [21]. Previous studies have not completely separated the data generation process from the inference process [22]. Our method requires only a single training session on a small dataset and does not necessitate the additional generation of pattern images during the inference phase. This efficiency is particularly advantageous when iterative inferences are required for a single mathematical model.

In the current implementation, we opted for NGBoost because the machine learning model must account for the uncertainty of predictions. Although NGBoost is a lightweight and excellent model, we have to determine the output format as a single parametric probability distribution. Therefore, if the predetermined probability distribution significantly differs from the true distribution, prediction accuracy may decrease. This is suggested by the results in the Supporting information(S6 Fig). To resolve this issue, the predictive model for parameters needs to be able to learn any probability distribution such as normalization flow [23].

This method can be applied to various pattern formation phenomena in life science. For example, although the Gray-Scott model is a famous pattern formation model, a good biological application has yet to be found. Our method will help discover an appropriate biological system to which we can apply this model. In addition, parameter estimation enables us to validate the models by comparing parameter space size and pattern frequency and by predicting pattern change following experimental perturbations. Confirming the effectiveness of this method through applications to other biological patterns is an important challenge for the future.

Given the constraints of data obtained from biological observations, we proposed the methods for screening candidate mathematical models and estimating specific parameters using only a limited number of snapshots. However, when multiple mathematical models can theoretically generate identical steady-state patterns—such as the Model B Cahn-Hilliard equation and the Model H advective Cahn-Hilliard equation, it may not be feasible to differentiate between them using steady-state data alone. In these cases, it becomes necessary to incorporate dynamic data, such as time-series information, for accurate model identification. Several promising approaches, including Physics-Informed Neural Network(PINN) [24] and Sparse Identification of Nonlinear Dynamics (SINDy) [2], have been developed to generate governing equations that are well-suited for scenarios where time-series data is available. These methods present viable options for dynamic data. Nonetheless, our parameter estimation approach also has the potential to be extended to such data. By applying NGBoost, implemented within SD-NPE, to data at arbitrary time points, it may be possible to resolve this challenge. However, SD-NPE is based on the assumption of independence between data points. Since time-series data inherently involves dependencies, the formation of a latent space that accounts for temporal information would be essential for accurate Bayesian estimation. Future work could focus on adapting SD-NPE to accommodate these dependencies, enabling it to effectively process dynamic data in a temporally-aware latent space.

The emphasis on both the deductive approach of mathematical models and the inductive approach of machine learning could compensate for each other’s weaknesses. This study offers opportunities for data-driven preliminary screening or validation of mathematical models. Mathematical models are well explainable but require theoretical understanding and domain-specific knowledge for their design. On the other hand, machine learning can provide accurate predictions that fit the data it has learned, though those are not explainable. Therefore, we thought the integration of these two methods was a significant issue in elucidating mechanisms that can accurately explain observational data in the field of mathematical biology. So far, there have been few such studies [25]. Through this research, we have presented a new direction in the analysis of mathematical models using machine learning.

## Limitations of the study

Preprocessing can significantly affect feature extraction into embedding vectors by CLIP. Without proper preprocessing, incorrect features can be caught by CLIP, and that may adversely affect model selection and parameter estimation. Currently, it is challenging to robustly extract information about abstract geometric patterns of interest from images in nature, and improvements are anticipated in future research.

Our method only applies to two-dimensional data, as the feature extraction process relies on the ViT model. To extend the method to three-dimensional data or other spatially structured data, such as spherical surfaces, ViT must be replaced with an appropriate encoder that supports these data formats.

Furthermore, there is a practical limit to the number of parameters that can be predicted due to computational constraints. We examined the relationship between the number of unknown parameters and the required training dataset size through parameter estimation in linear regression models (S2 Fig). The results suggest that the impact of the number of parameters on dataset size requirements is not substantial. However, as mentioned in the original NGBoost paper [7], the computational cost of the algorithm scales cubically with the number of parameters and linearly with the dataset size. This implies that executing the model becomes challenging when the number of parameters exceeds five. To address this limitation, an alternative estimator with a lower computational cost should be adopted.

## Supporting information

S1 Text Data generation by mathematical models. 1.1. Turing model. 1.2. Gray-Scott model. 1.3. Kernel-based Turing (KT) model. 1.4. Edwards-Wilkinson model. 1.5. Eden model. 1.6. DLA model. 1.7. L-system. 1.8. Phase-field model. 1.9. Cahn-Hilliard model.
(PDF)

S2 Text Theoretical derivation and validation of SD-NPE. 2.1. Deriviation. 2.2. Validation of parameter estimation.
(PDF)

S3 Text Redundancy of CLIP latent space for parameter estimation. Add descriptive text after the title of the item (optional).
(PDF)

S4 Text Procedure for calculating MAP@k. Add descriptive text after the title of the item (optional).
(PDF)

S5 Text Effect of image features on embedding into CLIP latent space. Add descriptive text after the title of the item (optional).
(PDF)

S1 Fig Validation of SD-NPE for simple linear regression models. (A) Comparison between analytical Bayesian inference and SD-NPE in the parameter space. Each figure illustrates the prior distribution and the changes in the posterior distribution as the sample size increases. (B) Comparison between analytical Bayesian inference and SD-NPE in the data space. In each figure, the linear function with the true parameters is indicated by a red line, the observed data points are shown as blue dots, the 95% confidence interval based on the posterior distribution from analytical Bayesian estimation is depicted by the shaded gray area, and the 95% confidence interval based on the posterior distribution obtained through SD-NPE is shown as the shaded red area. (C) An example of the relationship between sample size and KL divergence. (D) The KL divergence curves for 1000 test cases. Red line represents mean and blue region indicates ±1 standard deviation.
(TIF)

S2 Fig Relationship between the size of training data and KL divergence in parameter estimation of simple linear regression using SD-NPE. (A) shows the prediction error with estimation from 3 samples, and (B) shows the error from 20 samples. The horizontal axis represents the size of the data used to train the SD-NPE, while the vertical axis shows the average KL divergence between the predictions on the test data and the analytical solution. Both axes are on a logarithmic scale. The results are color-coded according to the number of unknown parameters in the linear regression problem.
(TIF)

S3 Fig Prediction examples for parameter estimation in a single-variable linear regression using SD-NPE for various dataset sizes. Each row corresponds to a dataset size used to train the SD-NPE: 10^3^, 10^4^, 10^5^, and 10^6^. For each dataset size, three examples are shown. The horizontal axis represents the predicted unknown parameter *a*, while the vertical axis indicates the probability density. The blue curve represents the probability distribution predicted by analytical Bayesian estimation, and the orange curve represents the approximate distribution generated by SD-NPE. The title above each plot indicates the KL divergence between these two distributions.
(TIF)

S4 Fig Prediction examples for parameter estimation in a two-variable linear regression using SD-NPE for various dataset sizes. Each row corresponds to a dataset size used to train the SD-NPE: 10^3^, 10^4^, 10^5^, and 10^6^. For each dataset size, three examples are shown. The horizontal and vertical axes represent the predicted unknown parameters *a* and *b*, respectively. The predicted parameter probability densities are shown using color gradients and contour lines. In each example, the left figure represents the results from analytical Bayesian estimation, while the right shows the predictions from SD-NPE. The title above each plot indicates the KL divergence between these two distributions.
(TIF)

S5 Fig Prediction examples for parameter estimation in a two-variable linear regression using SD-NPE, for various dataset sizes. Each row corresponds to a dataset size used to train the SD-NPE: 10^3^, 10^4^, 10^5^, and 10^6^. For each dataset size, three examples are shown. The vertical and horizontal axes represent the unknown variables *a* and *b*. For visualization purposes, the predicted probability distribution is shown by extracting the *ab* -plane where parameter *c* is set to its true value. The predicted parameter probability densities are shown using color gradients and contour lines. In each example, the left figure represents the results from analytical Bayesian estimation, while the right shows the predictions from SD-NPE. The title above each plot indicates the KL divergence between these two distributions.
(TIF)

S6 Fig Validation of SD-NPE for linear regression models with redundant parameters. (A) Comparison between analytical Bayesian inference and SD-NPE in the parameter space. Each figure illustrates the prior distribution and the changes in the posterior distribution as the sample size increases. (B) Comparison between analytical Bayesian inference and SD-NPE in the data space. In each figure, the linear function with the true parameters is indicated by a red line, the observed data points are shown as blue dots, the 95% confidence interval based on the posterior distribution from analytical Bayesian estimation is depicted by the shaded gray area, and the 95% confidence interval based on the posterior distribution obtained through SD-NPE is shown as the shaded red area. (C) An example of the relationship between sample size and KL divergence. (D) The KL divergence curves for 1000 test cases. Red line represents mean and blue region indicates ±1 standard deviation.
(TIF)

S7 Fig Cumulative explained variance curve of PCA to CLIP embedding vectors. The red lines show the point at which the cumulative contribution rate reaches 95%.
(TIF)

S8 Fig Pattern recognition ability of CLIP to stripe patterns. (A) Recognition of direction (top), boundary sharpness (middle), and wavenumber (bottom) of stripe patterns. Color indicates the image generation parameters *θ*, *a*, and *k*. (B) Box plots of mean nDCG in which each image generation parameter was selected as the rating criteria. The left plots (green) correspond to random sorting, and the right plots (brown) correspond to the sorting based on cosine similarity on CLIP latent space.
(TIF)

S9 Fig Pattern recognition ability of CLIP to Turing patterns. (A) Scatter plots of Turing patterns in our dataset on the feature space in which the horizontal axis is *k*_max_ and the vertical axis is *D*_k_. The markings correspond to Turing pattern images in (C). (B) Scatter plots on UMAP of CLIP latent space. The markings correspond to Turing pattern images in (C). (C) Examples of Turing pattern images with various values of *k*_max_ and *D*_k_. (D)(E) Scatter plots on UMAP of CLIP latent space. Colors indicates *k*_max_ (D) or *D*_k_ (E). (F) Box plots of mean nDCG in which *k*_max_ and *D*_k_ were selected as the rating criteria. The left plots (green) correspond to random sorting, and the right plots (brown) correspond to the sorting based on cosine similarity on CLIP latent space.
(TIF)

S10 Fig Analysis of embedding vectors of pattern images. (A) Clustering of pattern images in the feature space of UMAP. The horizontal and vertical axes represent the first and second components of the two-dimensional vectors obtained from UMAP, respectively.(B) The histogram and kernel density estimation of cosine similarity of each model.
(TIF)

S11 Fig Searching for similar pattern images for parameters and mathematical models not considered in the model selection dataset. Examples of the top five similar patterns are shown for target images with parameters outside the range specified when constructing the model selection dataset, as well as for mathematical models not included in the dataset. (A) Example with *g*_*v*_ = 2.5187, with other parameters set to *f*_*u*_ = 0.3692, *f*_*v*_ = 0.9572, *g*_*u*_ = 0.9812, *D*_*u*_ = 0.1, and *D*_*v*_ = 1.0. (B) Example with *D*_*v*_ = 10.0, with other parameters set to *f*_*u*_ = 0.0956, *f*_*v*_ = 0.0726, *g*_*u*_ = 0.9715, *g*_*v*_ = 0.5262, and *D*_*u*_ = 0.1. (C) Example with *D*_*u*_ = 0.01, with other parameters set to *f*_*u*_ = 0.2016, *f*_*v*_ = 0.6238, *g*_*u*_ = 0.4506, *g*_*v*_ = 0.6050, and *D*_*v*_ = 1.0. (D) Example of a pattern image generated by the Cahn-Hilliard model. The parameter is *γ* = 0.1.
(TIF)

S12 Fig Top 50 pattern images ranked by cosine similarity to the target image of Turing model. In the example shown in Fig 3B, which includes the target image and the top three most similar pattern images, the top 50 pattern images are also displayed.
(TIF)

S13 Fig The selection of mathematical models that can represent the skin pattern of humphead wrasse. (A) An image of humphead wrasse (*Cheilinus undulatus*). Left: original image. Center: cropped view of the red square region in the left image. Right: cropped view of the yellow square region in the center image, used as the target for model selection. (B) The target image after preprocessing and the top three images with the highest similarity. (C) Representation of cosine similarity to the target. Star shows the position of the target image. The horizontal and vertical axes represent the first and second components of the two-dimensional vectors obtained from UMAP, respectively. (D) The histogram of cosine similarity scores of all datasets to the target image. (E)The histogram and kernel density estimation of cosine similarity of each model.
(TIF)

S14 Fig The effect of crop size on the similarity ranking. The relationship between the image cropping applied as a preprocessing step in model selection and the top five most similar pattern images is illustrated using skin pattern of the humphead wrasse (*Cheilinus undulatus*) as an example.
(TIF)

S15 Fig Pattern images of Turing model on the parameter space. Each pattern image is one example generated by Turing model with the corresponding parameters. The horizontal axis represents *f*_*v*_, and the vertical axis represents *g*_*v*_. Patterns corresponding to the parameters in the bottom right area of the figure cannot be generated.
(TIF)

S16 Fig Training loss and MAP@*k* score of contrastive learning for the dimensionality reduction model. (A) Training loss was calculated for each batch. The blue line represents the raw values, while the red dashed line indicates the smoothed values. (B) MAP@*k* was calculated at the end of each epoch. The blue line represents the raw values, and the red dashed line indicates the smoothed values. The smoothing process was consistent with the method used in TensorBoard. A weighted average was calculated between the previous value and the current value in a 0.95: 0.05 ratio, and the result was divided by 1 − 0.95^*t*^ as a debiasing step, where *t* represents the number of data points up to the previous point.
(TIF)

S17 Fig Examples of parameter estimation of three models with different random seeds. SD-NPE was initialized with different random seeds to estimate the parameters *f*_*v*_ and *g*_*v*_ of the Turing model. The predictions are shown for five different parameter sets used as test cases for each model. The data size of training dataset was 2000 (A) and 10000 (B).
(TIF)
