# Supplementary material for: Data-driven discovery and parameter estimation of mathematical models in biological pattern formation
Source: PLoS Comput Biol. 2025 Jan 23;21(1):e1012689. doi: 10.1371/journal.pcbi.1012689 (PMC11756800; doi:10.1371/journal.pcbi.1012689)
Supplement: S1 Text — 1.1. Turing model. 1.2. Gray-Scott model. 1.3. Kernel-based Turing (KT) model. 1.4. Edwards-Wilkinson model. 1.5. Eden model. 1.6. DLA model. 1.7. L-system. 1.8. Phase-field model. 1.9. Cahn-Hilliard model. (PDF) [file pcbi.1012689.s001.pdf]

1 Data generation by mathematical models

1.1 Turing model

The Turing model is a mathematical model for pattern formation proposed by Alan Turing [1]. This model is particularly used to understand the mechanisms behind the formation of various natural patterns, such as animal coat markings and hair follicles, especially within a biological context [2]. The fundamental concept of the Turing model consists of two interacting morphogens: an activator and an inhibitor [3]. Spontaneous spatial patterns arise when the inhibitor diffuses more rapidly than the activator under certain conditions. We used following type of Turing system in our study:

$$\frac{\partial u}{\partial t} = f_u u - f_v v + qu^2 - u^3 + D_u \Delta u, \tag{9}$$

$$\frac{\partial v}{\partial t} = g_u u - g_v v + D_v \Delta v. \tag{10}$$

The parameters of the equations consist of the linear terms in the reaction component, f_u, f_v, g_u, g_v, the quadratic term q, and the diffusion coefficients D_u and D_v. For all data used in generating pattern images, the diffusion coefficients were set to $D_u = 0.1$ and $D_v = 1.0$. In constructing the model selection dataset, the parameters f_u,f_v,g_u, and g_v were randomly selected from a uniform distribution within the range $[0.1, 1.0]$. Additionally, the parameter q was randomly assigned a value of either 0 or 1. For the parameter estimation dataset, we fixed $f_u = 0.51$, $g_u = 0.81$, and $q = 0$, while f_v and g_v were the parameters to be predicted and randomly selected from a uniform distribution over the range $[0.6, 1.0]$. Images that did not reach a non-trivial steady state were excluded from the dataset.

In the numerical simulations, the spatial domain was discretized using the finite difference method, with periodic boundary conditions. The system's time integration was computed using the implicit method. Initial values in the spatial domain were assigned based on random numbers drawn from a uniform distribution over the range $[-0.5, 0.5]$. For the Turing model, the concentration of the component u was treated as a grayscale pattern image. The entire spatial domain of the array used in the calculations was adopted as the pattern image. When the simulation time was set to $T = 10000$, we observed in several cases that the standard deviation of the differences in the component u values for each pixel per a single iteration fell below 10^{-5}, indicating that the emerging patterns had reached a steady state. Therefore, the time $T = 10000$ was selected for data generation across all simulations of this model.

1.2 Gray-Scott model

The Gray-Scott model is a reaction-diffusion system that forms a variety of spatiotemporal patterns. This model describes the evolution of two chemical species, u, and v, which can react with each other and diffuse through a medium [4, 5].

This model assumes the following chemical reactions:

$$U + 2V \to 3V, \tag{11}$$

$$V \to P. \tag{12}$$

The model is defined by:

$$\frac{\partial u}{\partial t} = f(1 - u) - uv^2 + D_u \Delta u, \tag{13}$$

$$\frac{\partial v}{\partial t} = uv^2 - (f + k)v + D_v \Delta v, \tag{14}$$

where u and v are the concentrations of the chemical species, D_u and D_v are their respective diffusion coefficients, f is the feed rate of u, and k is the kill rate of v. For all data used in generating pattern images, the diffusion coefficients were set to $D_u = 0.00002$ and $D_v = 0.00001$. The parameter k was set within the range [0.03, 0.065] and f within [0.005, 0.06], with both parameters incremented in steps of 0.001. Images were excluded from the dataset if the concentration of u in space became uniform within a short period without forming characteristic pattern dynamics. To account for temporal variation in the patterns, multiple time points $[5000, 6250, 7500, 8750, 10000]$ were included in the dataset for each instance of data generation.

In the numerical simulations, the spatial domain was discretized using the finite difference method with periodic boundary conditions applied. The system's time integration was calculated using the explicit Euler method. The initial values in the spatial domain differed between the center and the peripheral. For the component u, each value in the central region was stochastically assigned from a uniform distribution in the range $[0.495, 0.505]$, while the peripheral values were set to 1.0. For the component v, each value in the central region was stochastically assigned from a uniform distribution in the range $[0.245, 0.255]$, with the peripheral values set to 0. For the Gray-Scott model, the concentration of the component u was treated as a grayscale pattern image. The entire spatial domain of the array used in the calculations was adopted as the pattern image. The grid spacing between pixels was $dx = 0.04$, and the time step for numerical simulation was $dt = 0.1$.

1.3 Kernel-based Turing (KT) model

KT model, introduced by S. Kondo, uses a convolution kernel and has the ability to generate a wider variation of patterns compared to the Turing system [6]. For example, It can form characteristic patterns like the caudal fin of guppy or the skin of Japanese common eel. The model is represented by the shape of an activation-inhibition kernel. The benefit of the model is that it can be implemented even when the detailed mechanism is not fully known. The model is defined by the following equations:

$$Kernel(x) = A(x) + I(x), \tag{15}$$

$$S(x) = \int u(x - x') * Kernel(x')dx', \tag{16}$$

$$\frac{\partial u}{\partial t} = S(x) - \deg * u, \tag{17}$$

where u is the concentration of a substance, $A(x)$ and $I(x)$ represent the positive and negative nonlocal spacial influences, respectively, and deg is the decomposition rate. In constructing the model selection dataset, both $A(x)$ and $I(x)$ were set to follow a Gaussian distribution according to the original KT model:

$$A(x) = \frac{A_{amp}}{\sqrt{2\pi}} \exp\left(-\frac{(x - \mu_A)^2}{2\sigma_A^2}\right), \tag{18}$$

$$I(x) = \frac{I_{amp}}{\sqrt{2\pi}} \exp\left(-\frac{(x - \mu_I)^2}{2\sigma_I^2}\right). \tag{19}$$

The parameter deg was set to 0.1. The parameters A_{amp}, μ_A, σ_A, I_{amp}, μ_I, and σ_I in the Eqs 18 and 19 were randomly selected from a uniform distribution within the range specified by Table S6 to generate pattern images. When the simulation time was set to $T = 30$, we observed in several cases that the standard deviation of the differences in the u values for each pixel per a single iteration fell below 10^{-2}, indicating that the

emerging patterns had reached a steady state. Therefore, the time $T = 30$ was selected for data generation. Images with spatial patterns that became uniform were excluded from the dataset based on the variance of the u concentration in space.

Table S6. Parameter set of KT model

Parameter	Value
A_{max}	[0,30]
μ_A	[0,10]
σ_A	[0,20]
I_{max}	[-30,0]
μ_I	[0,10]
σ_I	[0,20]

In the numerical simulations, the spatial domain was discretized using the finite difference method with periodic boundary conditions. Time integration of the system was calculated using the explicit Euler method. Initial values in the spatial domain were assigned randomly, with each pixel's value drawn from a uniform distribution in the range $[0, 1.0]$. The entire spatial domain of the array used in the calculations was adopted as the pattern image. The grid spacing between pixels was set to $dx = 1.56$, and the time step for numerical simulation was $dt = 0.1$. After computing Eq 16, the values of $S(x)$ were clipped to the range $[0, 2]$ to ensure computational stability.

1.4 Edwards-Wilkinson model

The Edwards-Wilkinson (E-W) model is a theoretical model used to describe the surface growth processes. It was introduced by S. F. Edwards and D. R. Wilkinson in 1982 as a way to understand the roughening of a surface under random deposition [7]. The E-W model is described by a linear stochastic differential equation:

$$\frac{\partial h}{\partial t} = \nu \Delta h + \eta, \tag{20}$$

where $h(x, t)$ represents the height of the surface at point x and time t, ν is the surface tension, and $\eta(x, t)$ is a noise term representing the random fluctuations in the growth process. This model is utilized to explain a fractal aspect of a skull suture [8] and scaling of the cell colony shape [9]. In constructing the model selection dataset, we extended the E-W model by incorporating nonlinear diffusion, as represented by the equations below:

$$\frac{\partial h}{\partial t} = \nu \Delta h^m + \eta, \tag{21}$$

$$\eta = \mathcal{N}(0, \sigma). \tag{22}$$

The exponent m of the diffusion term was selected from $[1, 1.5, 2]$ and the standard deviation σ of the noise term was selected from $[0.1, 0.55, 1.0]$. The dataset included multiple pattern images with identical parameters; however, the initial values and random seeds for the noise term were different.

In the numerical simulations, the spatial domain was discretized using the finite difference method with periodic boundary conditions applied. Time integration of the system was calculated using the explicit Euler method. The initial values in the spatial domain were set to zero for all pixels. The entire spatial domain of the array used in the calculations was adopted as the pattern image. The grid spacing between pixels was set to $dx = 1.0$, and the time step for numerical simulation was $dt = 0.1$. The time for data generation was set to $T = 100$.

1.5 Eden model

The Eden model is a stochastic growth model that is used to simulate the clonal growth of biological cells. It was proposed by Murray Eden in the 1960s to study the patterns that emerge during the colony growth of bacteria and other organisms [10]. In the Eden model, growth occurs by the random addition of identical cells to the outer perimeter of an existing cluster. Starting from an initial seed, at each time step, a new cell is added to a randomly chosen site that is adjacent to the cluster. The growth pattern is typically compact with a rough outer boundary. This model can be applied to the description of tumor growth [11]. The pseudocode for the algorithm used to generate clusters based on the Eden model is shown below (Algorithm 1).

Algorithm 1 Eden Model

```
Initialize a 2D grid G of size N × N
Initialize the cluster C as an empty list
Define the starting point S as the initial seed position (e.g., center of the grid)
Add S to the cluster C
while number of particles < maximum_particles do
    Define a list of potential new particles P, initially empty
    for each cell in the cluster C do
        for each neighboring cell N of this cell do
            if N is empty and not already in P then
                Add N to P
            end if
        end for
    end for
    Randomly select a particle p from P
    Add p to the cluster C
    Update the grid G to mark p as occupied
end while
Output the final cluster C
```

The shape of clusters generated by the Eden model is dependent on the initial condition S. The initial condition S was selected from two types: a center point in the grid or a vertical line passing through the center of the grid. The cluster sizes were set to various values, ranging approximately from 3×10^5 to 3×10^6 elements.

1.6 DLA model

The Diffusion-Limited Aggregation (DLA) model, introduced by T.A. Witten and L.M. Sander in 1983 [12], is a fractal growth model that simulates patterns formed through a process known as diffusion-limited aggregation. This model has been influential in the study of pattern formation in systems where diffusion is the primary constraint on growth. In the DLA model, particles undergo a random walk until they come into contact with a pre-existing aggregate and stick to it, leading to growth. The model starts with a seed particle, and subsequent particles are introduced far from the aggregate, diffusing randomly until they adhere to the cluster. This model has been utilized in coral reef growth [13] The pseudocode for the algorithm used to generate clusters based on the DLA model is shown below (Algorithm 2). The loop number was set to 2500 and attachment limit was set to 100000.

Algorithm 2 Diffusion Limited Aggregation Model

$r \leftarrow r\_init$
$flag \leftarrow False$
for $t \leftarrow 1$ to loop number **do**
    Generate initial particle position
    while particle is not attached to cluster **do**
        Randomly move particle
        if particle exceeds boundary or reaches attachment limit **then**
            Move on to the next iteration
        end if
    end while
    Attach particle to cluster (Update r accordingly)
end for

1.7 L-system

The L-system is one of the formal grammars for describing recursive structures introduced by the biologist Aristid Lindenmayer in 1968 [14], and applied to plant branching pattern [15]. We generated tree-structured patterns based on the L-system. The algorithm was described using pseudo code (Algorithm 3). At each branching point, the branch length was reduced by a factor of r_l, the thickness by a factor of r_t, and the direction was rotated by an angle of θ.

Algorithm 3 Tree structure by L-system

Require: $t_{\text{init}} > 0, \theta > 0, 0 < r_t \leq 1, 0 < r_l \leq 1$

$V_0 = \{(0, 1)\}$
Draw a line $\boldsymbol{v} = [0, 1]$ with the thickness of t_{init} from the origin point.

for $i = 1, 2, \ldots, \text{MaxDepth}$ **do**
    $t_i = t_{\text{init}} r_t^i$
    Sample r_d from a normal distribution $\mathcal{N}(0, \sigma)$
    Define rotation matrices with noise:

$$M_{\text{left}} = \begin{bmatrix} \cos(r_d\theta) & \sin(r_d\theta) \\ -\sin(r_d\theta) & \cos(r_d\theta) \end{bmatrix}, M_{\text{right}} = \begin{bmatrix} \cos(r_d\theta) & -\sin(r_d\theta) \\ \sin(r_d\theta) & \cos(r_d\theta) \end{bmatrix}$$

    $V_i = \{r_l(\boldsymbol{v} \cdot M_{\text{left}}), r_l(\boldsymbol{v} \cdot M_{\text{right}}) | \boldsymbol{v} \in V_{i-1}\}$
    Draw lines V_i with the thickness of t_i from the each end of the current tree.
end for

In constructing the model selection dataset, σ was fixed at 0.2 and r_t at 0.7. The parameters r_l and θ were varied in increments of 0.25 within the range [0.5, 0.9], with θ specified in radians. The initial branch thickness t_init was set to 5 when using the Python plotting library matplotlib. Note that this scale is different from that of the branch length. The tree-like figures generated with matplotlib were used as pattern images.

1.8 Phase-field model

The phase field model is a mathematical model used in materials science and physics to simulate the evolution of interfaces and shapes in a system undergoing phase transitions. It was developed as a tool to understand the formation of crystals [16]. The model uses a continuous field variable, typically denoted as u, to represent the state of the system. In this research, the model is defined by:

$$\frac{\partial u}{\partial t} = u(1-u)(u - 0.5 + \alpha(v - 0.5)) + \Delta u, \tag{23}$$

$$\frac{\partial v}{\partial t} = (1-u) - v + \Delta v, \tag{24}$$

where u represents some system state coupled with diffusible field v, and α is a constant that represents the efficacy of v on u dynamics. Phase field methods has been used to model single-cell shape [17] and skull sutre pattern formation [18]

In the numerical simulations, the spatial domain was discretized using the finite difference method with periodic boundary conditions applied. Time integration of the system was calculated using the explicit Euler method. The initial condition consists of a linear region containing noise, centered within the image. The details of the calculation used to determine the initial condition are provided in the pseudocode below (Algorithm 4). For the phase-field model, the concentration of the component u was treated as a grayscale pattern image. The entire spatial domain of the array used in the calculations was adopted as the pattern image. The grid spacing between pixels was set to $dx = 0.2$, and the time step for the numerical simulation was $dt = 0.05$. Pattern images at multiple time points $[100, 200, 300, 400, 500]$ were included in the dataset.

Algorithm 4 Initialization of phase-field model

Require: $grid\_size = 128$, u, v are 128×128 matrices
  for $x = 0$ to $grid\_size - 1$ **do**
    for $y = 0$ to $grid\_size - 1$ **do**
      $d = (y - grid\_size/2)^2$
      $u[x, y] = 1$ **if** $d < 100 + \mathcal{N}(0, 10)$ **else** 0
      $v[x, y] = 1 - u[x, y]$
    end for
  end for

1.9 Cahn-Hilliard model

The Cahn-Hilliard equation, named after John W. Cahn and John E. Hilliard, describes the time evolution of spinodal decomposition [19]. This equation is used as a model for polymer mixtures and alloys. Since it generates spatial patterns similar to those of the Turing and KT models over time, we employed it solely for validation in S11 Fig. The equation is shown below:

$$\frac{\partial u}{\partial t} = D\Delta(u^3 - u - \gamma \Delta u). \tag{25}$$

The parameter γ, representing the length of the transition region between the two phases, was set to $\gamma = 0.1$ and the diffusion coefficient was set to $D = 1.0$. In the numerical simulations, the spatial domain was discretized using the finite difference method with periodic boundary conditions. Time integration of the system was calculated using the explicit Euler method. Initial values in the spatial domain were

assigned based on random numbers drawn from a uniform distribution over the range [-0.1,0.1]. The entire spatial domain of the array used in the calculations was adopted as the pattern image. Based on empirical observations, the simulation time was set to $T = 5$. The time step used in the simulation was $dt = 0.0001$, and the spatial resolution was $dx = 0.25$.

References

1. Mathison TA. The chemical basis of morphogenesis. Phil Trans R Soc Lond B. 1952;237(641):37-72.

2. Murray JD. Mathematical Biology II: Spatial Models and Biomedical Applications. 3rd ed. Springer New York, NY; 2003.

3. Gierer A, Meinhardt H. A theory of biological pattern formation. Kybernetik. 1972;12(1):30–39.

4. Gray P, Scott S. Autocatalytic reactions in the isothermal, continuous stirred tank reactor: isolas and other forms of multistability. Chemical Engineering Science. 1983;38(1):29-43.

5. Pearson JE. Complex patterns in a simple system. Science. 1993;261(5118):189–192.

6. Kondo S. An updated kernel-based Turing model for studying the mechanisms of biological pattern formation. Journal of Theoretical Biology. 2017;414:120–127.

7. Edwards SF, Wilkinson D. The surface statistics of a granular aggregate. Proceedings of the Royal Society of London A Mathematical and Physical Sciences. 1982;381(1780):17–31.

8. Naroda Y, Endo Y, Yoshimura K, Ishii H, Ei SI, Miura T. Noise-induced scaling in skull suture interdigitation. PLOS ONE. 2020;15:1-14.

9. Oguma T, Takigawa-Imamura H, Miura T. Mechanism underlying dynamic scaling properties observed in the contour of spreading epithelial monolayer. Phys Rev E. 2020;102:062408.

10. Eden M. A two-dimensional growth process. Dynamics of fractal surfaces. 1961;4:223–239.

11. Junior SCF, Martins ML, Vilela MJ. A growth model for primary cancer. Physica A: Statistical Mechanics and its Applications. 1998;261(3):569-580.

12. Witten TA, Sander LM. Diffusion-limited aggregation. Physical review B. 1983;27(9):5686.

13. Kaandorp JA. Macroscopic Modelling of Environmental Influence on Growth and Form of Sponges and Corals Using the Accretive Growth Model. ISRN Biomathematics. 2013;2013.

14. Aristid L. Mathematical models for cellular interactions in development I. Filaments with one-sided inputs. Journal of Theoretical Biology 1968; 18(3):280-299.

15. Prusinkiewicz P. Graphical applications of L-systems. Proceedings of graphics interface. 1986;86(86):247–253.

16. Kobayashi R. Modeling and numerical simulations of dendritic crystal growth. Physica D: Nonlinear Phenomena. 1993;63(3):410–423.

17. Nonomura M. Study on Multicellular Systems Using a Phase Field Model. PLOS ONE. 2012;7:1–9.

18. Miura T, Perlyn CA, Kinboshi M, Ogihara N, Kobayashi-Miura M, Morriss-Kay GM, et al. Mechanism of skull suture maintenance and interdigitation. Journal of Anatomy. 2009;215(6):642–655.

19. Cahn JW, Hilliard JE. Free Energy of a Nonuniform System. I. Interfacial Free Energy. The Journal of Chemical Physics. 1958;28(2):258-267
