# Supplementary material for: Data-driven discovery and parameter estimation of mathematical models in biological pattern formation
Source: PLoS Comput Biol. 2025 Jan 23;21(1):e1012689. doi: 10.1371/journal.pcbi.1012689 (PMC11756800; doi:10.1371/journal.pcbi.1012689)
Supplement: S2 Text — 2.1. Deriviation. 2.2. Validation of parameter estimation. (PDF) [file pcbi.1012689.s002.pdf]

2 Theoretical derivation and validation of SD-NPE

2.1 Derivation

The feature vector extracted from the image is denoted as x, and the parameters for the mathematical model are denoted as y. Then the NGBoost training dataset D consisting of N samples is expressed as follows:

$$D = \{(x_1, y_1), (x_2, y_2), \cdots, (x_N, y_N)\}. \tag{26}$$

The process by the NGBoost model is expressed as $y = f(x)$. When described in Bayesian notation, y can be expressed as $p(y \mid x)$. Thus, The prior distribution of the parameters is:

$$\begin{aligned} p(y) &= \int p(y \mid x) p(x) dx \qquad (27) \\ &= \int f(x) p(x) dx. \qquad (28) \end{aligned}$$

Since D is constructed from images generated based on parameters stochastically determined according to a specified probability distribution, it can be approximated by Monte Carlo integration as:

$$p(y) = \int f(x) p(x) dx \;\approx\; \frac{1}{N} \sum_{x_i \in D} f(x_i). \tag{29}$$

Therefore, the prior distribution learned by NGBoost can be computed by averaging the output of NGBoost across all samples.

Consider the set of samples X that are generated by the same parameter set y for n times:

$$X = \{x_1, x_2, \cdots, x_n\}. \tag{30}$$

Using the trained NGBoost, we demonstrate that the posterior distribution of parameters $p(y \mid X)$ can be obtained. According to Bayes' theorem:

$$p(y \mid X) = \frac{p(X \mid y) p(y)}{p(X)}. \tag{31}$$

Applying the mean field approximation to $p(X \mid y)$ results in:

$$p(y \mid X) = \prod_{x_i \in X} p(x_i \mid y) \frac{p(y)}{p(X)}. \tag{32}$$

Using Bayes' theorem for $p(x \mid y)$, we get:

$$p(y \mid X) = \prod_{x_i \in X} \frac{p(y \mid x_i) p(x_i)}{p(y)} \cdot \frac{p(y)}{p(X)}. \tag{33}$$

Since $p(x)$ and $p(X)$ are constants independent of y, we denote the terms involving them as k as follows:

$$k = \frac{\prod_{x_i \in X} p(x_i)}{p(X)}. \tag{34}$$

The posterior distribution $p(y|X)$ is then:

$$p(y \mid X) = k \prod_{x_i \in X} \frac{p(y|x_i)}{p(y)} \cdot p(y) \tag{35}$$

$$= k \prod_{x_i \in X} \frac{f(x_i)}{p(y)} \cdot p(y). \tag{36}$$

Herein, the probability distribution $p(y)$ was derived utilizing the trained NGBoost model, as described in Eq 29. Although the constant k is unknown, since the parameter space satisfies the following constraint, the value of k can be determined by numerically integrating $p(y \mid X)$ over a finite interval of possible solutions in the parameter space:

$$\int p(y \mid X) dy = 1, \tag{37}$$

$$k = \frac{1}{\int \prod_{x_i \in X} \frac{f(x)}{p(y)} \cdot p(y) dy}. \tag{38}$$

Therefore, the posterior distribution of the parameters can be approximated using the NGBoost process $f(x)$ as follows:

$$p(y \mid X) = \frac{\prod_{x_i \in X} \frac{f(x)}{p(y)} \cdot p(y)}{\int \prod_{x_i \in X} \frac{f(x)}{p(y)} \cdot p(y) dy}. \tag{39}$$

2.2 Validation of parameter estimation

We verified that the SD-NPE in the previous section closely approximates the analytically obtained Bayesian inference.

Initially, we considered the following linear regression model with explanatory variable x and target variable y:

$$y = a + bx + \epsilon, \tag{40}$$

where a and b are the model parameters and ϵ represents the noise of this model. These model parameters and noise are subject to the following equations:

$$\boldsymbol{w} = \left[\begin{smallmatrix} a \\ b \end{smallmatrix}\right] = \mathcal{N}(\boldsymbol{\mu}_0, \boldsymbol{\Sigma}_0), \tag{41}$$

$$\boldsymbol{\mu}_0 = \left[\begin{smallmatrix} 0 \\ 0 \end{smallmatrix}\right], \boldsymbol{\Sigma}_0 = \left[\begin{smallmatrix} 1,0 \\ 0,1 \end{smallmatrix}\right], \tag{42}$$

$$\epsilon = \mathcal{N}(0, 0.2), \tag{43}$$

where we represented the model parameters a and b as the column vector $\boldsymbol{w}$. When preparing the data, the values of x were randomly sampled from a continuous uniform distribution over the interval $[-1, 1]$.

In this linear regression problem, the posterior distribution of $\boldsymbol{w}$ can be analytically formulated [1]. Since the prior distribution is the two-dimensional standard normal distribution, the posterior is also normally distributed. Let the input of the obtained data set be denoted as $X = \{x_1, \ldots, x_N\}$ and the corresponding target variables as $Y = \{y_1, \ldots, y_N\}$. Then, we represented the target variables $\{y_n\}$ collectively as the column vector $\boldsymbol{y}$ and defined accuracy parameter as $\beta = (1/0.2)^2 = 25$ based on the variance of the noise ϵ and the design matrix $\boldsymbol{\Phi}$ as follows:

$$\boldsymbol{\Phi} = \begin{pmatrix} 1 & x_1 \\ 1 & x_2 \\ \vdots & \vdots \\ 1 & x_N \end{pmatrix}. \tag{44}$$

Here, the posterior distribution is expressed as:

$$p(\boldsymbol{w}|\boldsymbol{y}) = \mathcal{N}(\boldsymbol{w}|\boldsymbol{\mu}_N, \boldsymbol{\Sigma}_N), \tag{45}$$

where

$$\boldsymbol{\mu}_N = \boldsymbol{\Sigma}_N(\boldsymbol{\Sigma}_0^{-1}\boldsymbol{\mu}_0 + \beta\boldsymbol{\Phi}^{\mathrm{T}}\boldsymbol{y}), \tag{46}$$

$$\boldsymbol{\Sigma}_N^{-1} = \boldsymbol{\Sigma}_0^{-1} + \beta\boldsymbol{\Phi}^{\mathrm{T}}\boldsymbol{\Phi}. \tag{47}$$

We compared the results of SD-NPE with the above true posterior distribution. NGBoost was trained using 10^5 samples of data consisting of points on the xy plane obtained according to the linear regression model and the corresponding true labels of $\boldsymbol{w}$. The output of NGBoost is a parametric probability distribution, and it is necessary to specify the type of distribution when training. A multivariate normal distribution was specified as the output format. Averaging the output of NGBoost across all samples yielded an approximate prior distribution of the learned parameters. The true prior distribution and this distribution were compared in S1A Fig. In one particular instance with 20 observed data points, the KL divergence value, which measures the magnitude of the difference between the two distributions, was 0.05206 indicating that the approximation was highly accurate.

The uncertainty of the predicted model parameters is reduced as the number of samples increases. The relationship between the number of samples and the estimation was shown in S1A and S1B Fig. In one example, the distribution of parameters which is estimated from only one sample by the present method deviated slightly from the analytical,and the KL divergence was more than 0.1. On the other hand, when there are multiple samples, the estimated distribution is sufficiently approximated (S1C Fig). We calculated the KL divergence of 1000 examples (S1D Fig). The parameters a, b of the data were sampled respectively in the interval [-2,2]. It is clear that the approximation accuracy of the present method increases with the number of samples. In this case, the approximation accuracy becomes a plateau when using more than 6 samples.

In addition, to investigate the relationship between the number of unknown parameters and the size of the training dataset required to achieve sufficient accuracy in SD-NPE, we also evaluated linear regression models with one and three unknown parameters. The model for a single unknown parameter is given as follows:

$$y = ax + \epsilon, \tag{48}$$

$$w = a = \mathcal{N}(\mu_0, \Sigma_0), \tag{49}$$

$$\mu_0 = 0, \Sigma_0 = 1, \tag{50}$$

$$\epsilon = \mathcal{N}(0, 0.2). \tag{51}$$

For the analytical derivation of the probability distribution, the design matrix is defined as follows:

$$\boldsymbol{\Phi} = \begin{pmatrix} x_1 \\ x_2 \\ \vdots \\ x_N \end{pmatrix}. \tag{52}$$

Also, the model for three unknown parameters is given as follows:

$$y = a + bx^{(1)} + cx^{(2)} + \epsilon, \tag{53}$$

$$\boldsymbol{w} = \begin{bmatrix} a \\ b \\ c \end{bmatrix} = \mathcal{N}(\boldsymbol{\mu}_0, \boldsymbol{\Sigma}_0), \tag{54}$$

$$\boldsymbol{\mu}_0 = \begin{bmatrix} 0 \\ 0 \end{bmatrix}, \boldsymbol{\Sigma}_0 = \begin{bmatrix} 1,0,0 \\ 0,1,0 \\ 0,0,1 \end{bmatrix}, \tag{55}$$

$$\epsilon = \mathcal{N}(0, 0.2). \tag{56}$$

The design matrix is defined as follows:

$$\boldsymbol{\Phi} = \begin{pmatrix} 1 & x_1^{(1)} & x_1^{(2)} \\ 1 & x_2^{(1)} & x_2^{(2)} \\ 1 & x_3^{(1)} & x_3^{(2)} \\ \vdots & \vdots & \\ 1 & x_N^{(1)} & x_N^{(2)} \end{pmatrix}. \tag{57}$$

Eqs 46 and 47 are similarly applicable in this case. We used dataset sizes of 10^3, 10^4, 10^5, and 10^6. For each dataset size and number of parameters, SD-NPE training was conducted three times, and the average KL divergence was used as the score, plotted as a double-logarithmic graph (S2 Fig). We evaluated cases with 3 and 20 test samples as inputs, keeping all other test conditions the same as previously described. Regardless of the sample size, we observed a trend in which the dataset size required to reduce KL divergence increased with the number of unknown parameters. However, because KL divergence is not well-suited for direct comparison across different prediction conditions, these scores should be interpreted with caution. To qualitatively assess the relationship between dataset size and prediction accuracy, we provided several examples for each number of parameters (S3, S4 and S5 Figs). With a dataset size of 10,000, outputs approximately matching the analytical results were obtained across all the number of parameters. While it is indeed necessary to increase the dataset size as the number of parameters grows, the results suggest that an exponential increase is not required.

Next, we examined the case where the parameters are not uniquely determined. Consider the following linear regression model:

$$y = (a + b)x + \epsilon, \tag{58}$$

where $\boldsymbol{w}$ and ϵ are also subject to the above Eqs 41, 42, and 43. In this problem, the values of a,b are not uniquely determined, and the result of Bayesian inference becomes a band-like shape in the parameter space. Note that a multivariate normal distribution was specified as the output format of NGBoost though the posterior distribution is not a typical parametric distribution. The prior distribution obtained by approximation through Monte Carlo integration was found to well approximate the true prior distribution(S6A Fig). The value of the KL divergence was 0.00031715. The results of the present method exhibit distributions that extend in band-like shapes, similar to the results of the analytical. While the probability in the periphery of this band-shaped distribution of the present method was estimated lower than the analytical, the overall features of the distribution are consistent(S6A Fig). The prediction error may be due to the multivariate normal distribution as the output format of NGBoost. As with the previous simple case, we quantified the relationship between the number of samples and the KL divergence(S6B-D Fig). In this case, the KL divergence of the present method did not decrease regardless of the number of samples. These results suggest the high

applicability of the present method to mathematical models. This is due to the fact that parameters of mathematical models are often redundant with respect to the characteristics of the generated spatial pattern, making it possible that parameters cannot be uniquely determined.

References

1. Bishop CM. Pattern Recognition and Machine Learning. 1st ed. Springer New York, NY. 2006.
