# Supplementary material for: Data-driven discovery and parameter estimation of mathematical models in biological pattern formation
Source: PLoS Comput Biol. 2025 Jan 23;21(1):e1012689. doi: 10.1371/journal.pcbi.1012689 (PMC11756800; doi:10.1371/journal.pcbi.1012689)
Supplement: S3 Text — Add descriptive text after the title of the item (optional). (PDF) [file pcbi.1012689.s003.pdf]

3 Redundancy of CLIP latent space for parameter estimation

To confirm the need for dimensionality reduction, We performed principal component analysis (PCA) on CLIP embedding vectors corresponding to pattern images generated by changing all parameters (i.e. f_u,f_v,g_u,g_v, and q) of the Turing model. Then we calculated the cumulative contribution ratio (S7 Fig). The top 100 principal components account for more than 95 percent of the variance between the embedding vectors. Therefore, the space required to describe the features of Turing patterns is significantly smaller than the entire latent space of CLIP (S7 Fig red line).
