# Supplementary material for: Data-driven discovery and parameter estimation of mathematical models in biological pattern formation
Source: PLoS Comput Biol. 2025 Jan 23;21(1):e1012689. doi: 10.1371/journal.pcbi.1012689 (PMC11756800; doi:10.1371/journal.pcbi.1012689)
Supplement: S4 Text — Add descriptive text after the title of the item (optional). (PDF) [file pcbi.1012689.s004.pdf]

4 Procedure for calculating MAP@k

To quantify the similarity of mathematical models in the CLIP latent space, we used Mean Average Precision at k (MAP@k). MAP@k is commonly used as an evaluation metric in information retrieval and recommendation tasks. It measures the precision of relevant items within the top k ranked results, providing insight into both the accuracy and ranking quality of the retrieved items in relation to a query. The method for calculating MAP is described below:

$$\text{MAP@}k = \frac{1}{N}\sum_{q=1}^{N} \text{AP}_q\text{@}k, \tag{59}$$

where

$$\text{AP}_q\text{@}k = \frac{1}{k}\sum_{i=1}^{k} P_q(i)\cdot \text{rel}_q(i), \tag{60}$$

$$P_q(i) = \frac{1}{i}\sum_{j=1}^{i} \text{rel}_q(j). \tag{61}$$

In this study, N represents the size of the dataset for the mathematical models designated as the target of model selection, and q denotes the index of a pattern image. The term $\text{rel}_q(j)$ indicates the relevance of the mathematical model that generates the pattern image ranked j-th in terms of similarity to the target model. $\text{rel}_q(j)$ can only take a value of 0 or 1, and the value of 1 signifies an appropriate recommendation for the target, whereas 0 indicates otherwise. In the MAP@50 score matrix (Fig 4), relevance is 1 only when the mathematical model specified by the column label is recommended. $P_q(i)$ represents the precision of the similarity rankings from the top result down to the i-th ranked prediction. $\text{AP}_q\text{@}k$ is the average of the precision calculated sequentially from the first to the k-th rank. Finally, MAP@k is the mean of $\text{AP}_q\text{@}k$ across the N-item dataset.
