# Supplementary material for: Data-driven discovery and parameter estimation of mathematical models in biological pattern formation
Source: PLoS Comput Biol. 2025 Jan 23;21(1):e1012689. doi: 10.1371/journal.pcbi.1012689 (PMC11756800; doi:10.1371/journal.pcbi.1012689)
Supplement: S5 Text — Add descriptive text after the title of the item (optional). (PDF) [file pcbi.1012689.s005.pdf]

5 Effect of image features on embedding into CLIP latent space

To confirm CLIP pattern recognition ability, we mapped two types of patterns, simple stripe patterns, and the Turing patterns, in CLIP latent space and examined the tendencies associated with some image features(S8 Fig). At first, we prepared the 2D stripe patterns based on the following formula:

$$u(x) = 1/(1 + e^{-a\sin(2\pi k\frac{x}{r})}), \tag{62}$$

where r represents the length of one side of the image, and k is a parameter that determines the periodicity of the striped pattern. The parameter a determines the sharpness of the stripes. The angles of shapes were changed with a continuous value of [0,180] degrees. In CLIP latent space, the embedding vectors of stripe patterns were distributed continuously and vertically according to the angle (S8A Fig). The distribution was also clearly positioned based on sharpness. The patterns were sharper on the left side. The parameter k, frequency of stripe patterns, was reflected in the lateral direction of the distribution. We quantified the sensitivity of CLIP to image features using the Normalized Documented Cumulative Gain (nDCG) metric, which is often used to measure the effectiveness of information retrieval algorithms, especially in the context of search engines and recommendation systems. To calculate the nDCG, we selected one image from all stripe patterns and sorted the CLIP embedding vectors according to the cosine similarity with that image. Then, the nDCG was calculated using one of image features (angle, sharpness and frequency) as an rating index. This operation was repeated for all images. The formula for nDCG at a particular rank position P can be written as:

$$\text{nDCG@}P = \frac{\text{DCG@}P}{\text{IDCG@}P}, \tag{63}$$

where

$$\text{DCG@}P = \sum_{i}^{P} \frac{rel_i}{\log_2(i+1)}, \tag{64}$$

and

$$\text{IDCG@}P = \sum_{i}^{|\text{REL@}P|} \frac{rel_i}{\log_2(i+1)}. \tag{65}$$

Here, rel_i represents the rating index of the item at position i, and $|REL@P|$ is the set of rating indices sorted in descending order up to position P. We set P to the number of all images. We compared the nDCG of CLIP with that of random sort as a control (S8B Fig). For all three features, nDCG of CLIP was clearly higher than the control. Thus, CLIP can recognize various features of images and embed the images into the latent space.

Next, we applied the same analysis to the Turing pattern. The image features of the Turing pattern, $K_{\max}$ and D_{k}, were continuously distributed on the CLIP latent space (S9A-E Fig). The nDCG of CLIP about $K_{\max}$ and D_{k} was also higher than the control. According to these results, CLIP is suitable for feature extraction from pattern images.
