# Supplementary figures and images for: Data-driven discovery and parameter estimation of mathematical models in biological pattern formation
Source: PLoS Comput Biol. 2025 Jan 23;21(1):e1012689. doi: 10.1371/journal.pcbi.1012689 (PMC11756800; doi:10.1371/journal.pcbi.1012689)

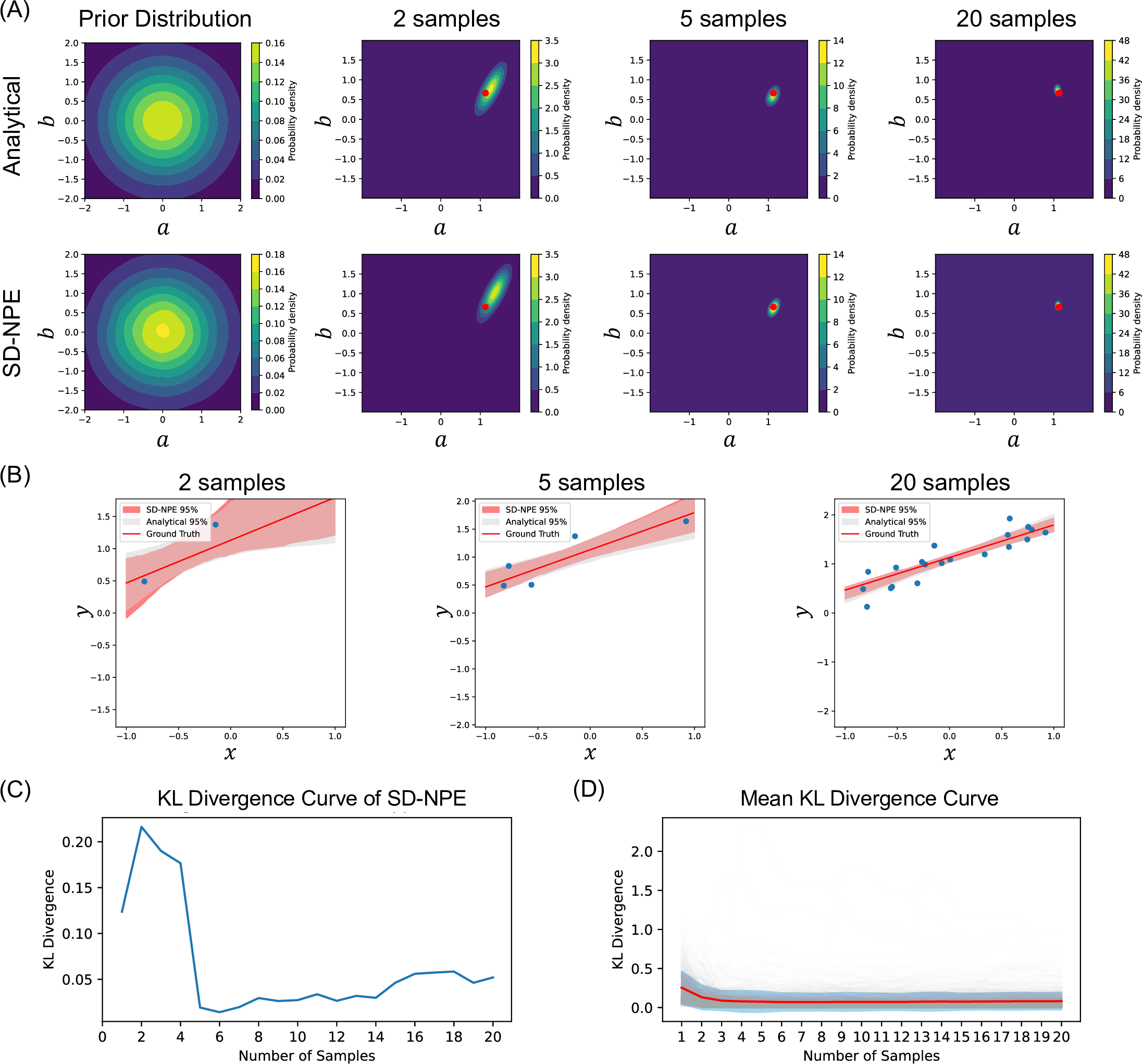

Supplement: S1 Fig — (A) Comparison between analytical Bayesian inference and SD-NPE in the parameter space. Each figure illustrates the prior distribution and the changes in the posterior distribution as the sample size increases. (B) Comparison between analytical Bayesian inference and SD-NPE in the data space. In each figure, the linear function with the true parameters is indicated by a red line, the observed data points are shown as blue dots, the 95% confidence interval based on the posterior distribution from analytical Bayesian estimation is depicted by the shaded gray area, and the 95% confidence interval based on the posterior distribution obtained through SD-NPE is shown as the shaded red area. (C) An example of the relationship between sample size and KL divergence. (D) The KL divergence curves for 1000 test cases. Red line represents mean and blue region indicates ±1 standard deviation. (TIF) [file pcbi.1012689.s006.tif]

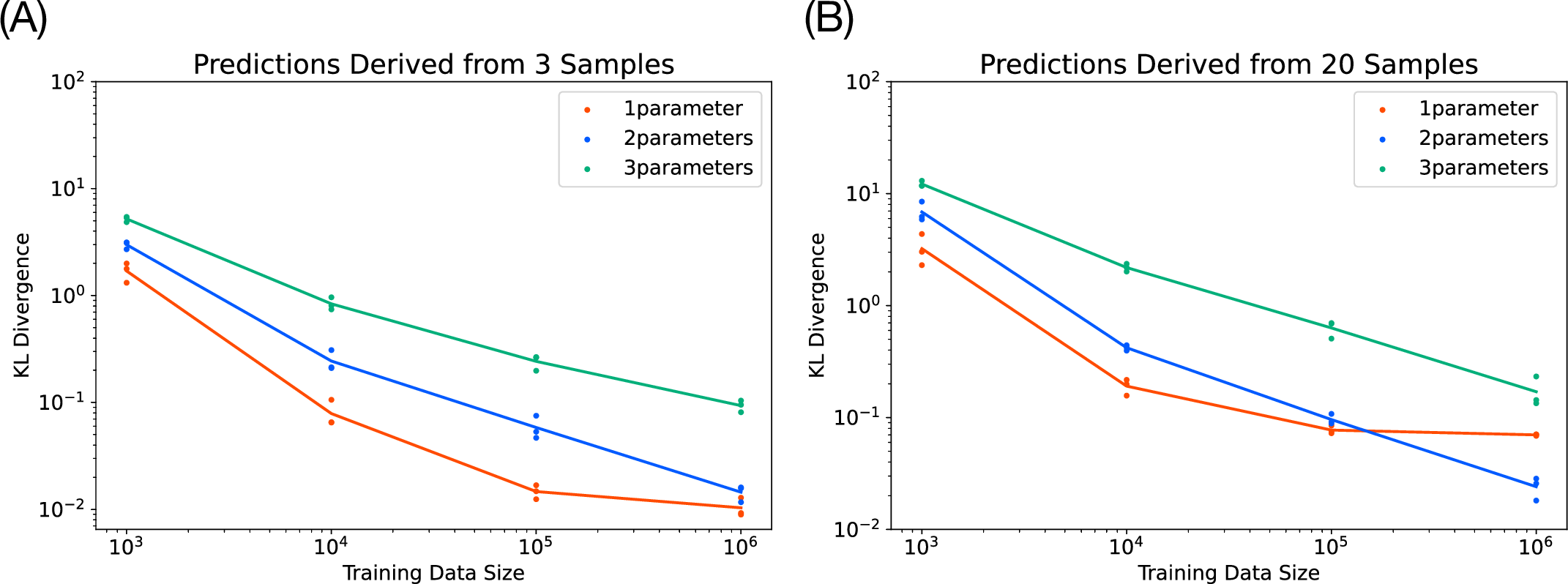

Supplement: S2 Fig — (A) shows the prediction error with estimation from 3 samples, and (B) shows the error from 20 samples. The horizontal axis represents the size of the data used to train the SD-NPE, while the vertical axis shows the average KL divergence between the predictions on the test data and the analytical solution. Both axes are on a logarithmic scale. The results are color-coded according to the number of unknown parameters in the linear regression problem. (TIF) [file pcbi.1012689.s007.tif]

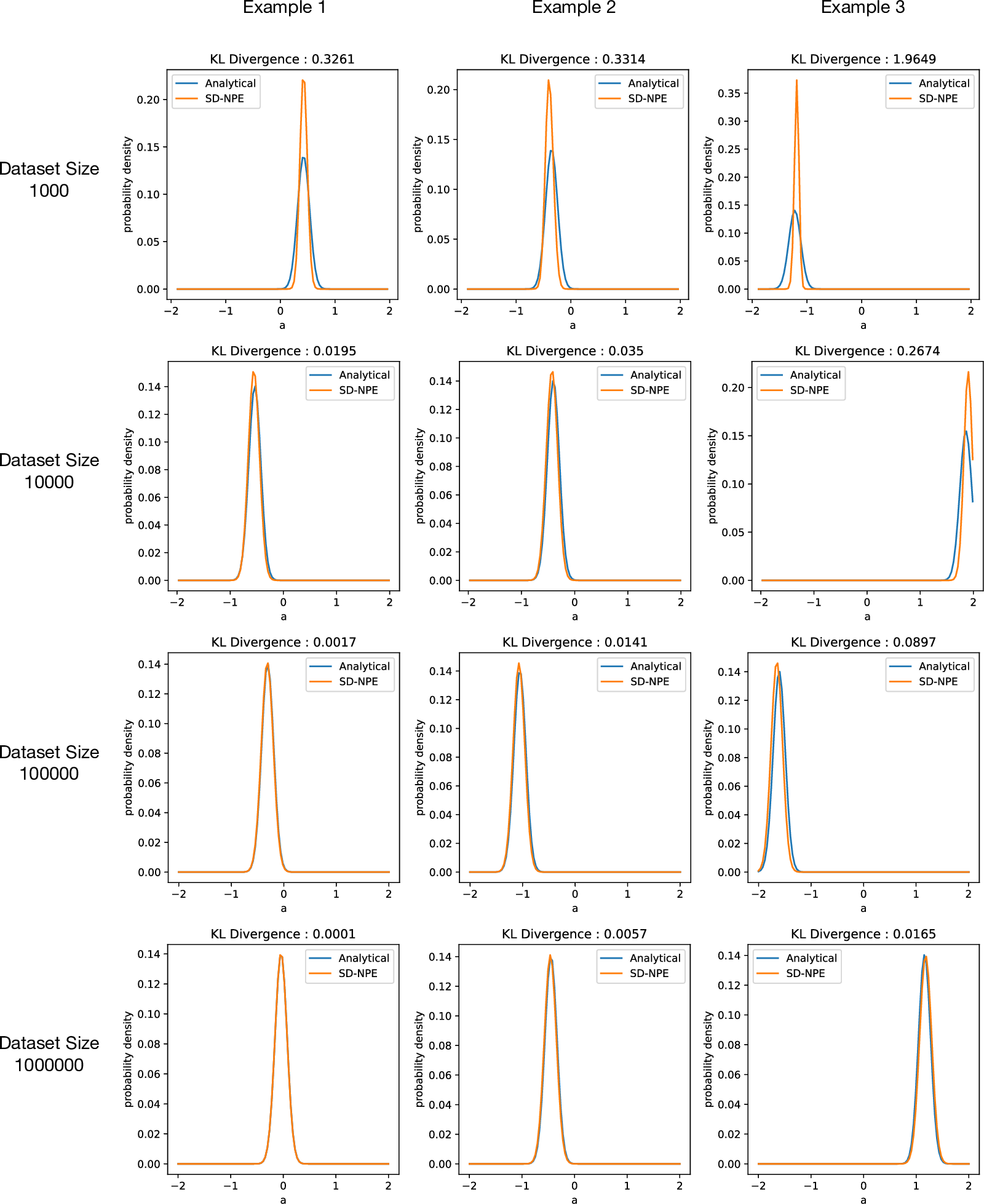

Supplement: S3 Fig — Each row corresponds to a dataset size used to train the SD-NPE: 103, 104, 105, and 106. For each dataset size, three examples are shown. The horizontal axis represents the predicted unknown parameter a, while the vertical axis indicates the probability density. The blue curve represents the probability distribution predicted by analytical Bayesian estimation, and the orange curve represents the approximate distribution generated by SD-NPE. The title above each plot indicates the KL divergence between these two distributions. (TIF) [file pcbi.1012689.s008.tif]

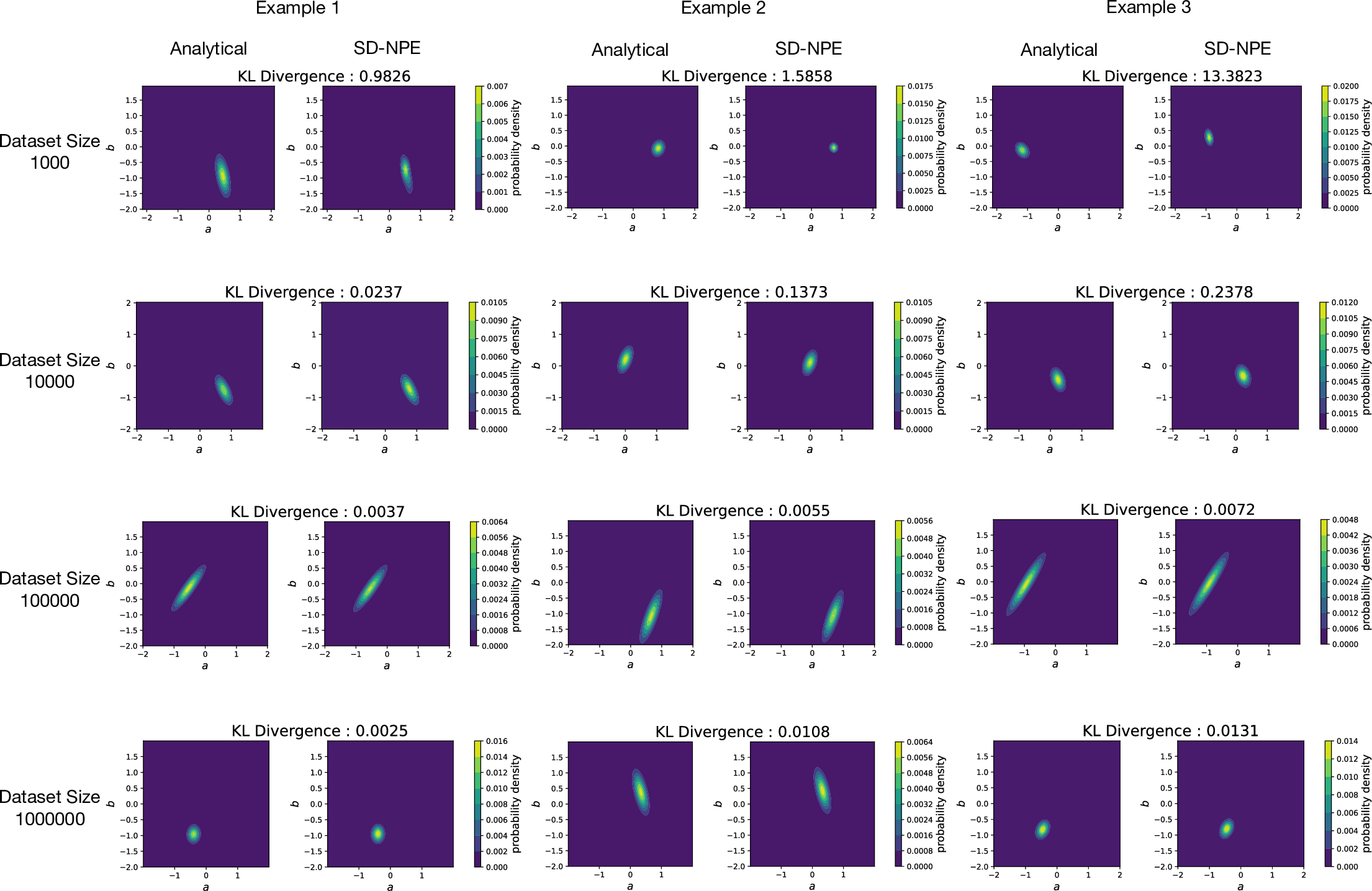

Supplement: S4 Fig — Each row corresponds to a dataset size used to train the SD-NPE: 103, 104, 105, and 106. For each dataset size, three examples are shown. The horizontal and vertical axes represent the predicted unknown parameters a and b, respectively. The predicted parameter probability densities are shown using color gradients and contour lines. In each example, the left figure represents the results from analytical Bayesian estimation, while the right shows the predictions from SD-NPE. The title above each plot indicates the KL divergence between these two distributions. (TIF) [file pcbi.1012689.s009.tif]

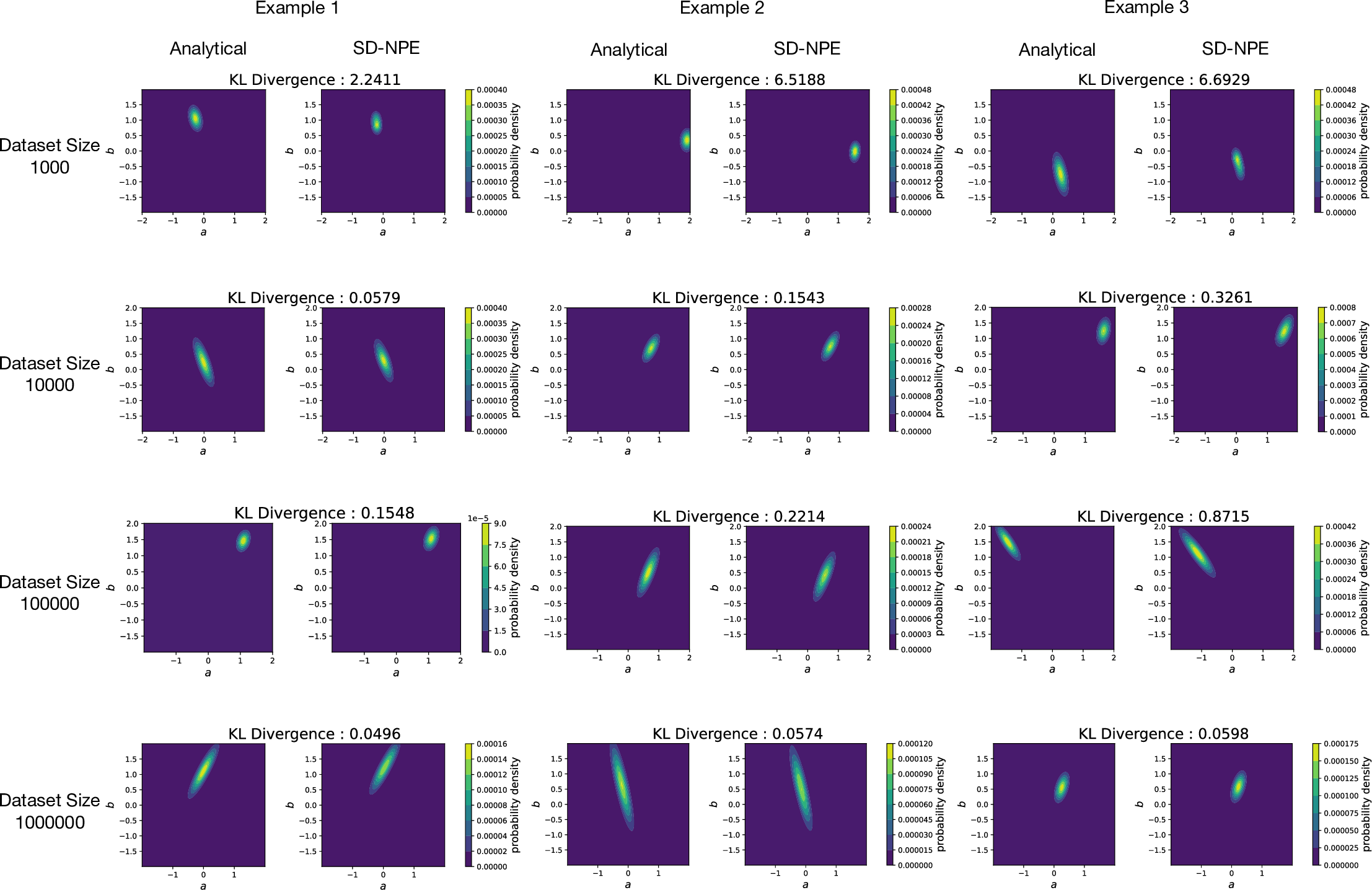

Supplement: S5 Fig — Each row corresponds to a dataset size used to train the SD-NPE: 103, 104, 105, and 106. For each dataset size, three examples are shown. The vertical and horizontal axes represent the unknown variables a and b. For visualization purposes, the predicted probability distribution is shown by extracting the ab -plane where parameter c is set to its true value. The predicted parameter probability densities are shown using color gradients and contour lines. In each example, the left figure represents the results from analytical Bayesian estimation, while the right shows the predictions from SD-NPE. The title above each plot indicates the KL divergence between these two distributions. (TIF) [file pcbi.1012689.s010.tif]

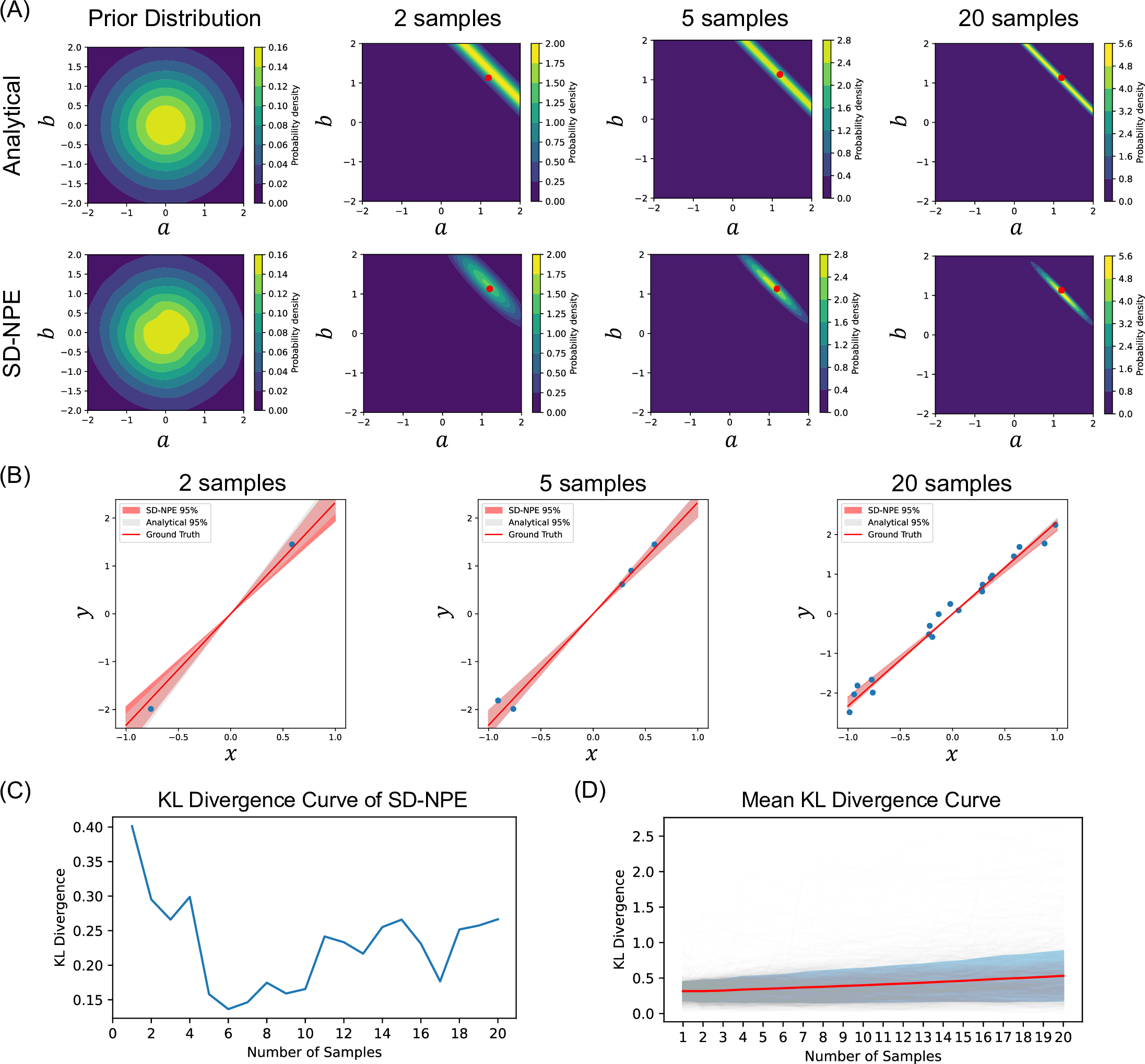

Supplement: S6 Fig — (A) Comparison between analytical Bayesian inference and SD-NPE in the parameter space. Each figure illustrates the prior distribution and the changes in the posterior distribution as the sample size increases. (B) Comparison between analytical Bayesian inference and SD-NPE in the data space. In each figure, the linear function with the true parameters is indicated by a red line, the observed data points are shown as blue dots, the 95% confidence interval based on the posterior distribution from analytical Bayesian estimation is depicted by the shaded gray area, and the 95% confidence interval based on the posterior distribution obtained through SD-NPE is shown as the shaded red area. (C) An example of the relationship between sample size and KL divergence. (D) The KL divergence curves for 1000 test cases. Red line represents mean and blue region indicates ±1 standard deviation. (TIF) [file pcbi.1012689.s011.tif]

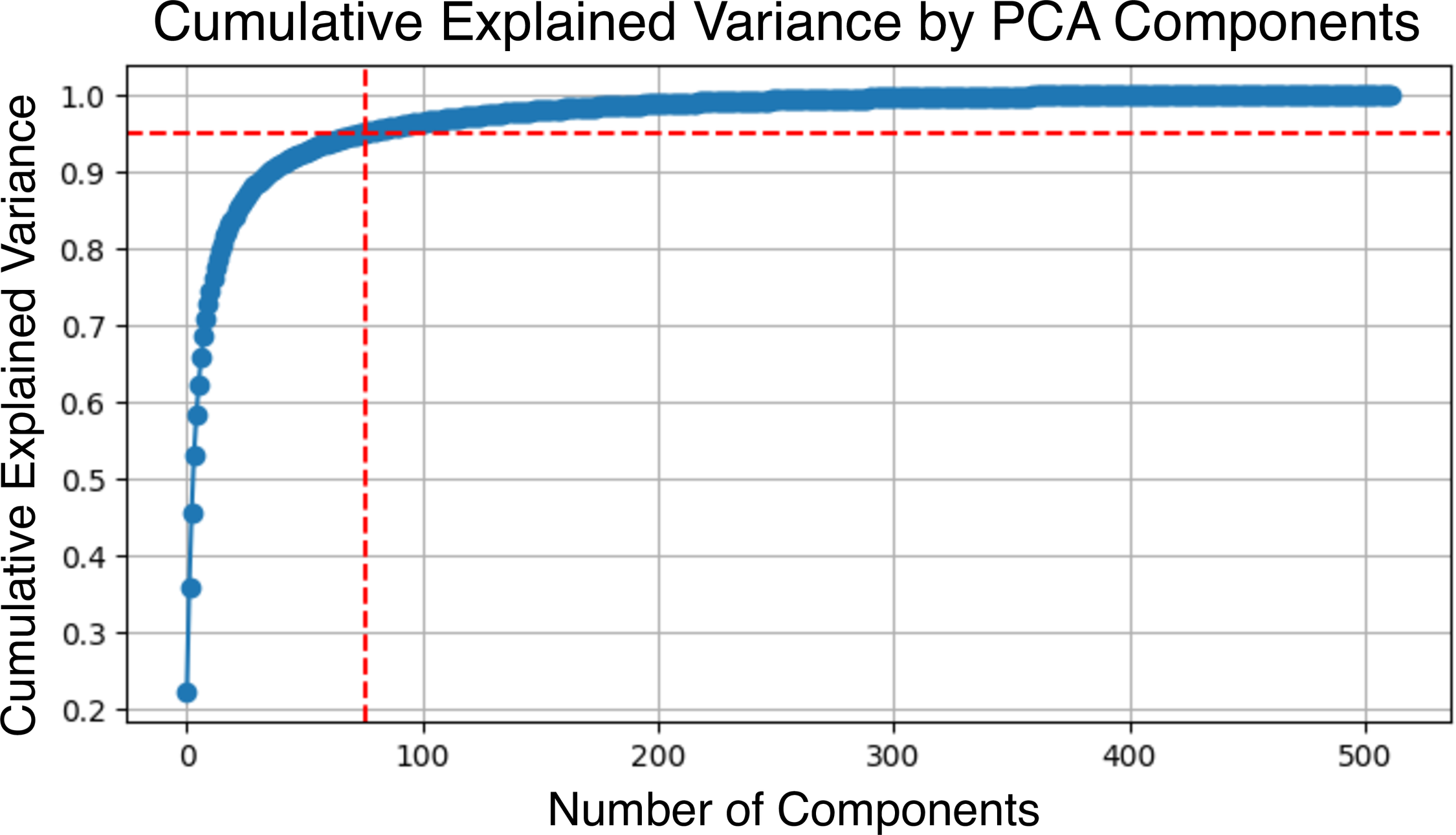

Supplement: S7 Fig — The red lines show the point at which the cumulative contribution rate reaches 95%. (TIF) [file pcbi.1012689.s012.tif]

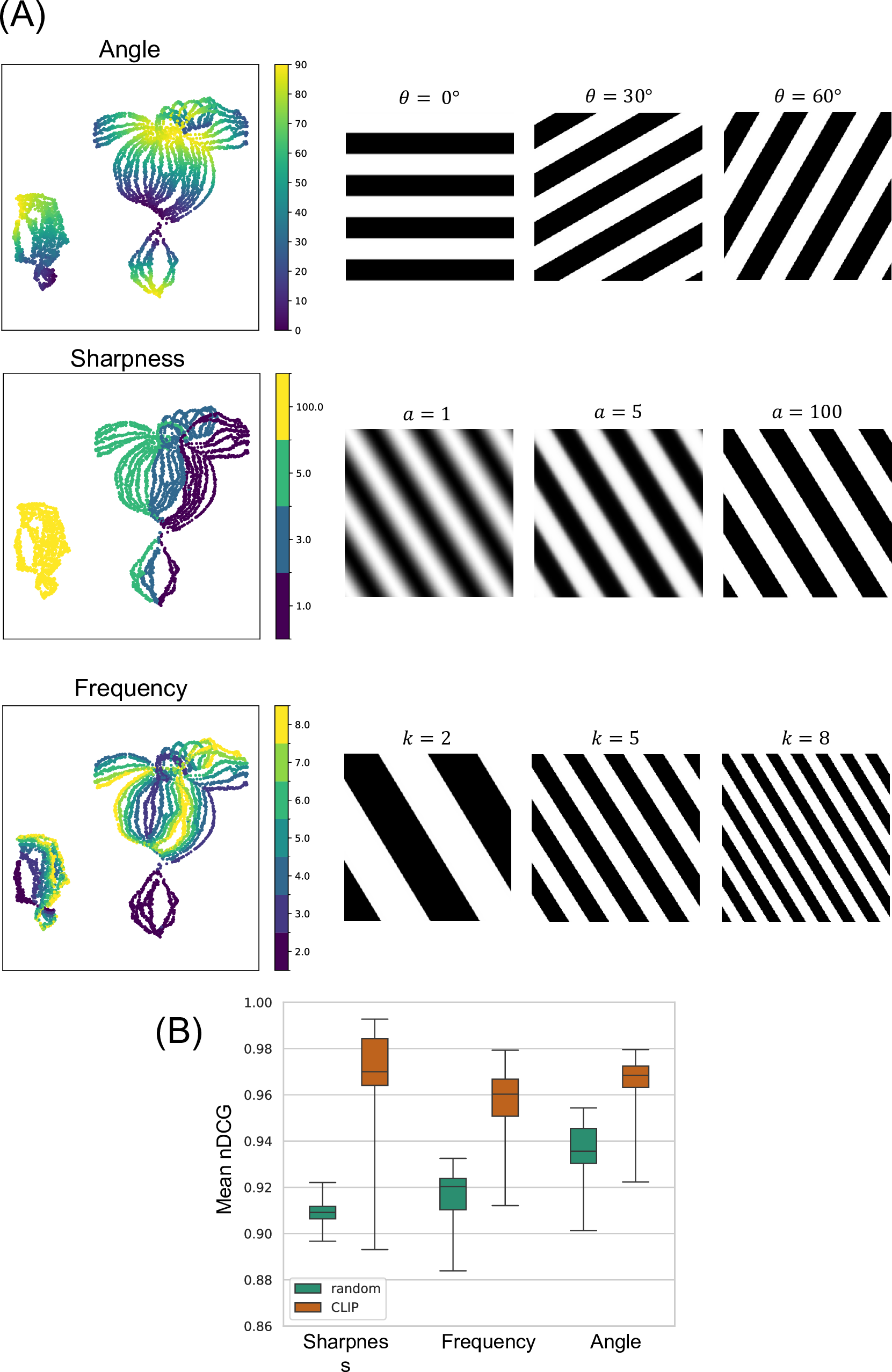

Supplement: S8 Fig — (A) Recognition of direction (top), boundary sharpness (middle), and wavenumber (bottom) of stripe patterns. Color indicates the image generation parameters θ, a, and k. (B) Box plots of mean nDCG in which each image generation parameter was selected as the rating criteria. The left plots (green) correspond to random sorting, and the right plots (brown) correspond to the sorting based on cosine similarity on CLIP latent space. (TIF) [file pcbi.1012689.s013.tif]

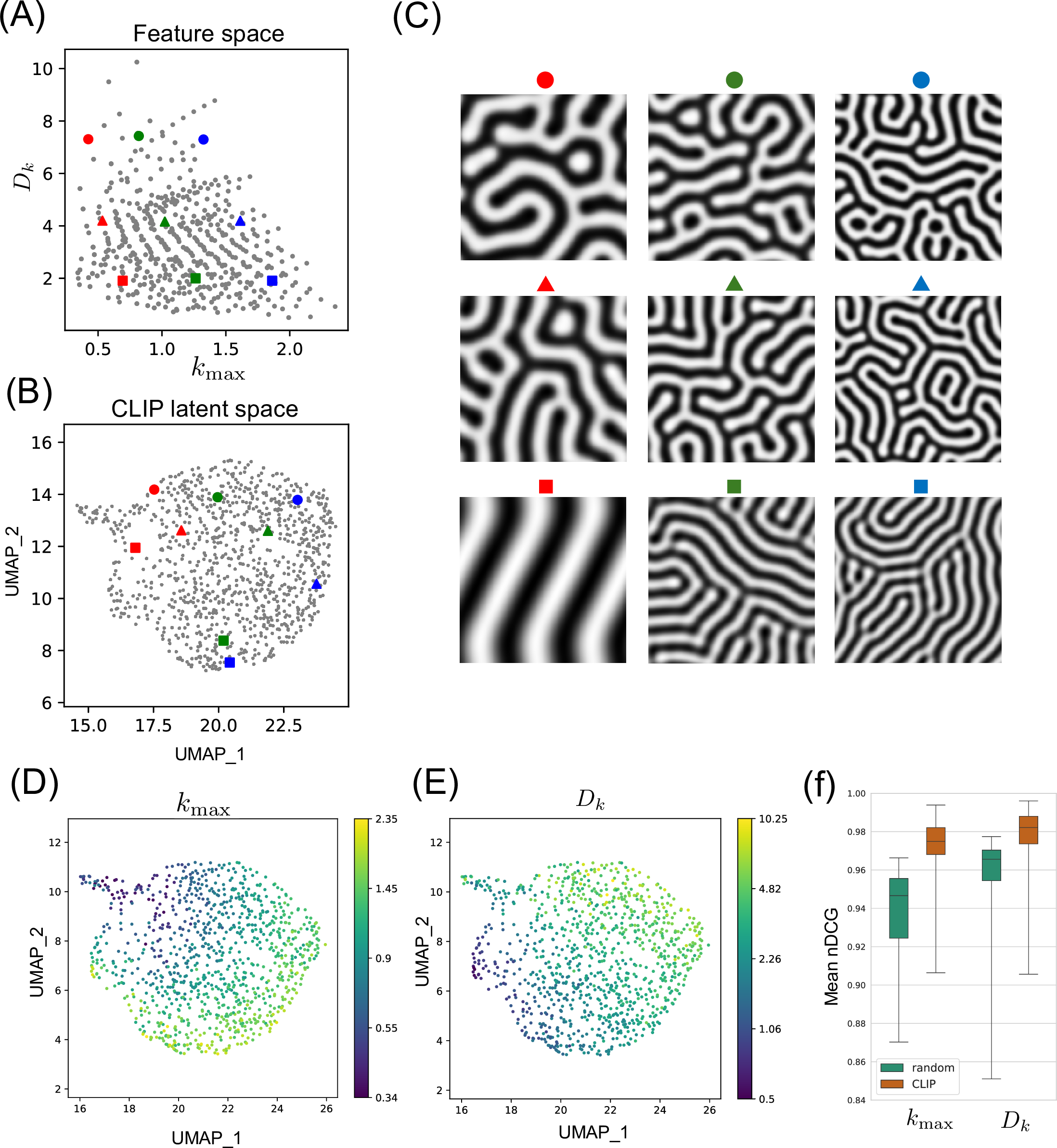

Supplement: S9 Fig — (A) Scatter plots of Turing patterns in our dataset on the feature space in which the horizontal axis is kmax and the vertical axis is Dk. The markings correspond to Turing pattern images in (C). (B) Scatter plots on UMAP of CLIP latent space. The markings correspond to Turing pattern images in (C). (C) Examples of Turing pattern images with various values of kmax and Dk. (D)(E) Scatter plots on UMAP of CLIP latent space. Colors indicates kmax (D) or Dk (E). (F) Box plots of mean nDCG in which kmax and Dk were selected as the rating criteria. The left plots (green) correspond to random sorting, and the right plots (brown) correspond to the sorting based on cosine similarity on CLIP latent space. (TIF) [file pcbi.1012689.s014.tif]

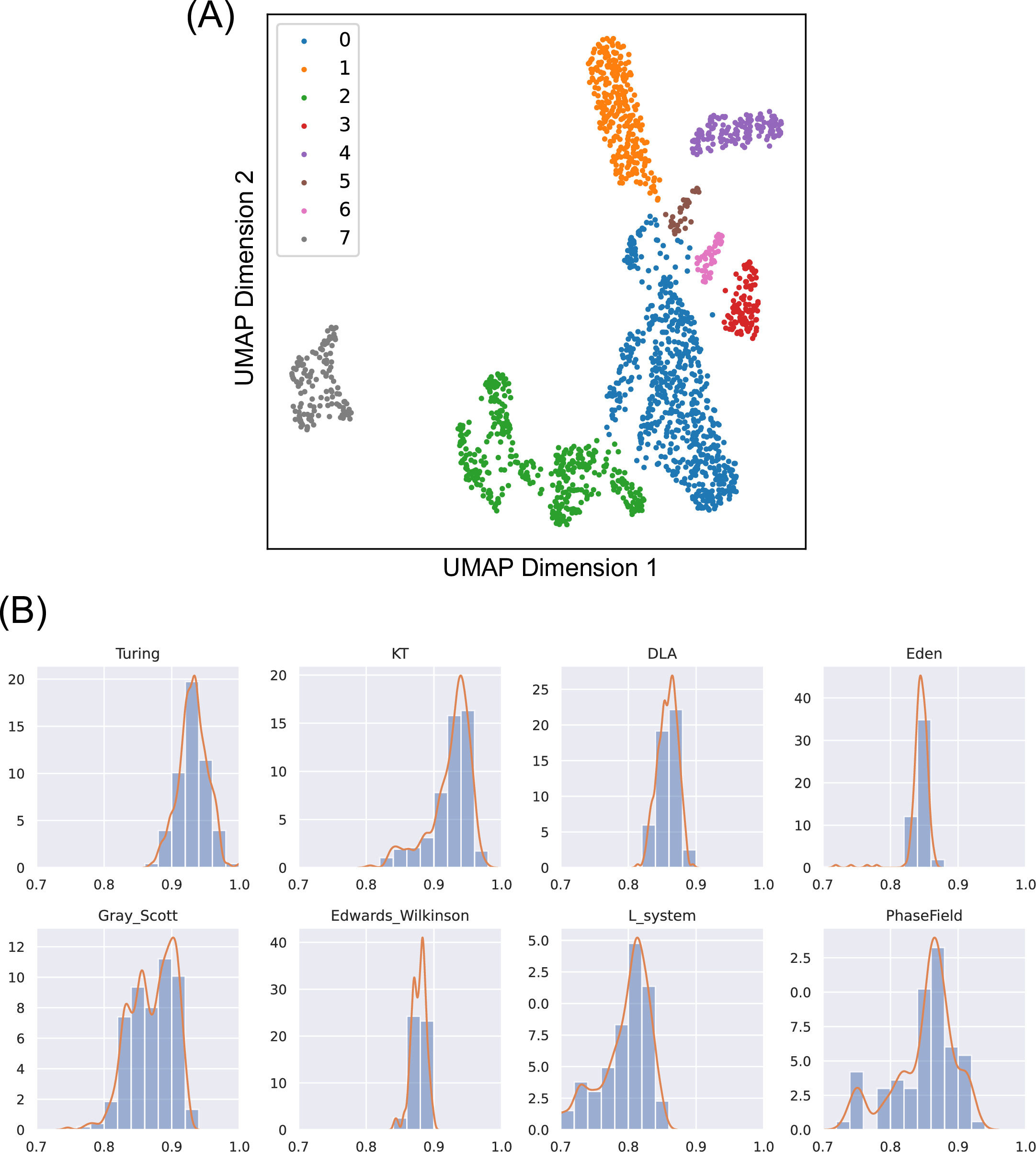

Supplement: S10 Fig — (A) Clustering of pattern images in the feature space of UMAP. The horizontal and vertical axes represent the first and second components of the two-dimensional vectors obtained from UMAP, respectively.(B) The histogram and kernel density estimation of cosine similarity of each model. (TIF) [file pcbi.1012689.s015.tif]

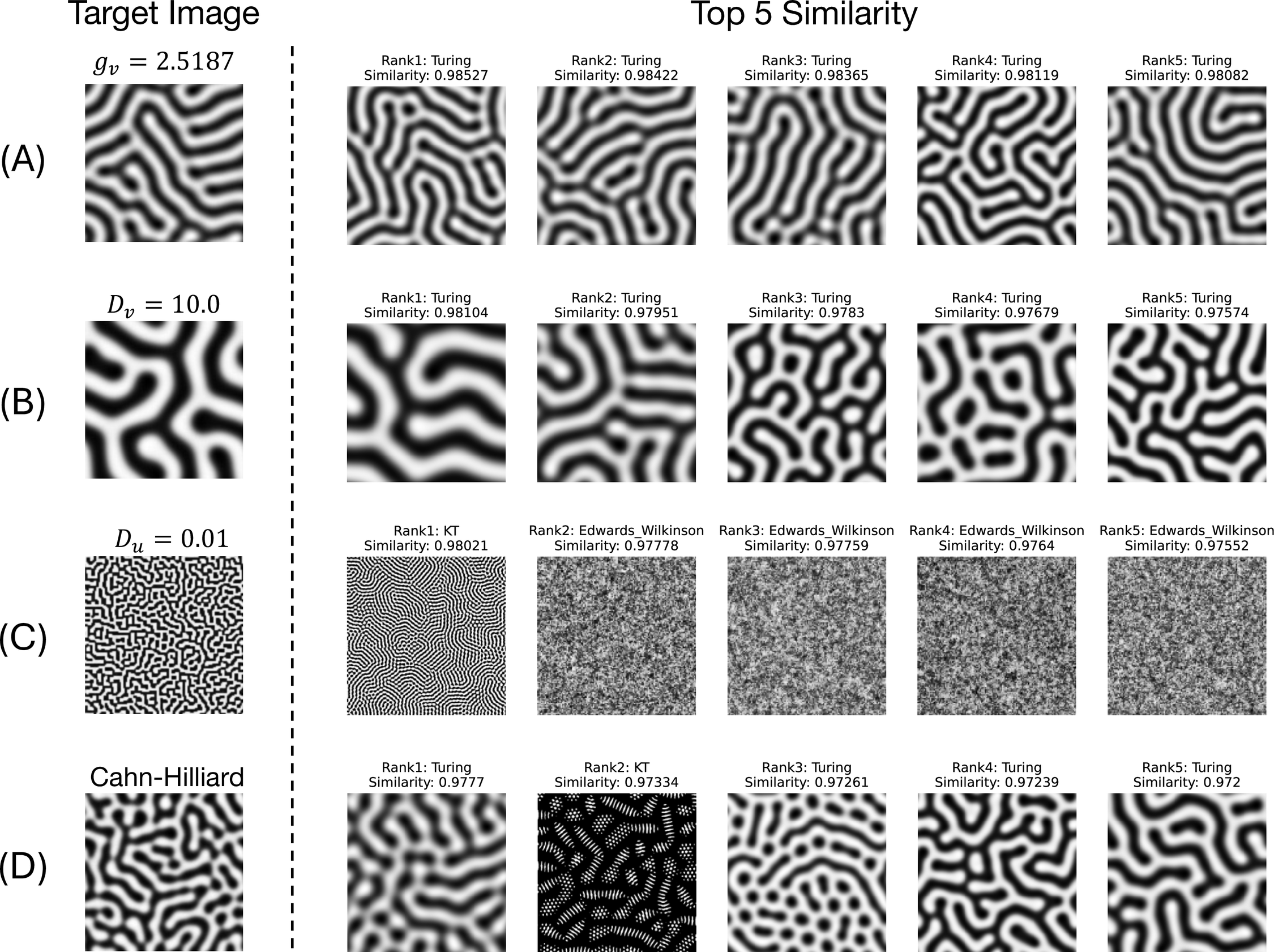

Supplement: S11 Fig — Examples of the top five similar patterns are shown for target images with parameters outside the range specified when constructing the model selection dataset, as well as for mathematical models not included in the dataset. (A) Example with gv = 2.5187, with other parameters set to fu = 0.3692, fv = 0.9572, gu = 0.9812, Du = 0.1, and Dv = 1.0. (B) Example with Dv = 10.0, with other parameters set to fu = 0.0956, fv = 0.0726, gu = 0.9715, gv = 0.5262, and Du = 0.1. (C) Example with Du = 0.01, with other parameters set to fu = 0.2016, fv = 0.6238, gu = 0.4506, gv = 0.6050, and Dv = 1.0. (D) Example of a pattern image generated by the Cahn-Hilliard model. The parameter is γ = 0.1. (TIF) [file pcbi.1012689.s016.tif]

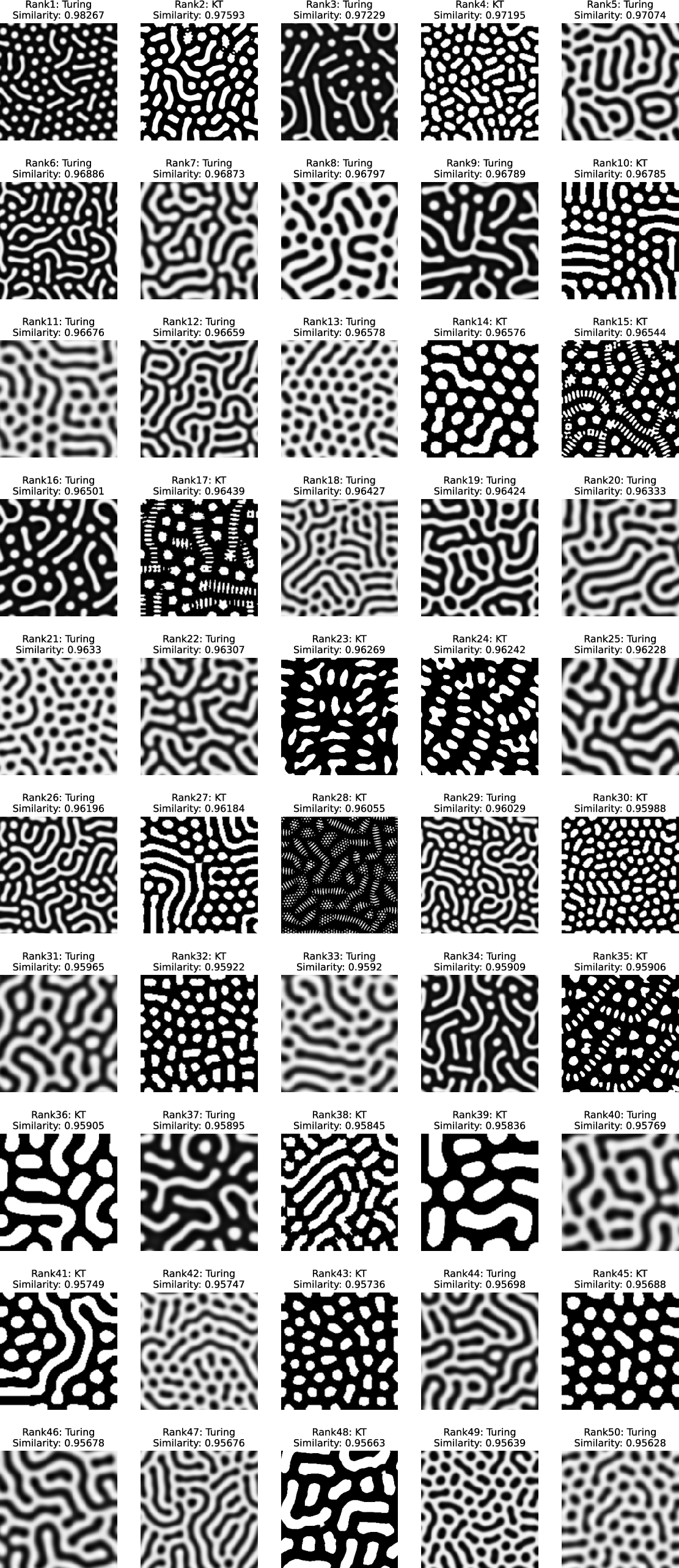

Supplement: S12 Fig — In the example shown in Fig 3B, which includes the target image and the top three most similar pattern images, the top 50 pattern images are also displayed. (TIF) [file pcbi.1012689.s017.tif]

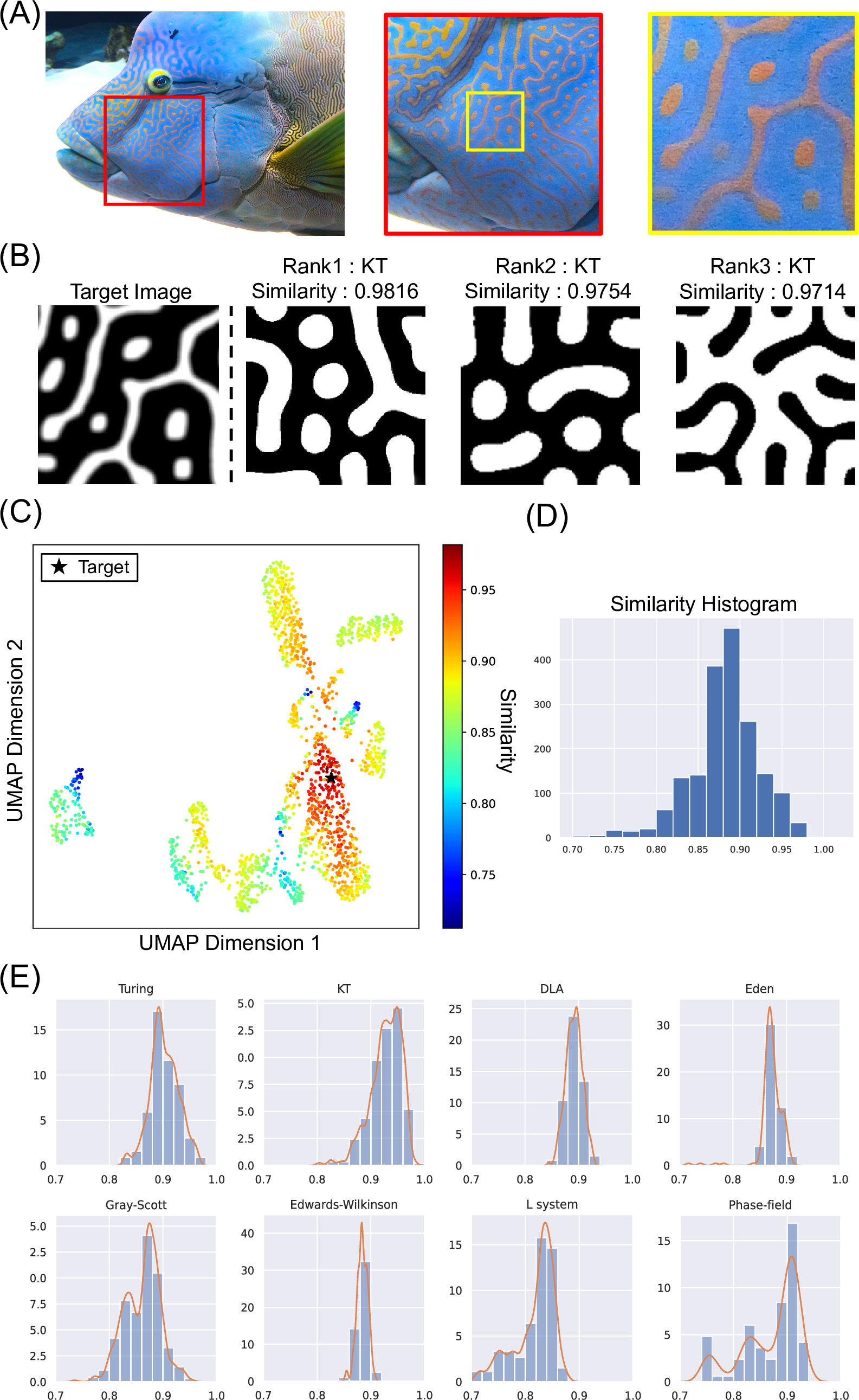

Supplement: S13 Fig — (A) An image of humphead wrasse (Cheilinus undulatus). Left: original image. Center: cropped view of the red square region in the left image. Right: cropped view of the yellow square region in the center image, used as the target for model selection. (B) The target image after preprocessing and the top three images with the highest similarity. (C) Representation of cosine similarity to the target. Star shows the position of the target image. The horizontal and vertical axes represent the first and second components of the two-dimensional vectors obtained from UMAP, respectively. (D) The histogram of cosine similarity scores of all datasets to the target image. (E)The histogram and kernel density estimation of cosine similarity of each model. (TIF) [file pcbi.1012689.s018.tif]

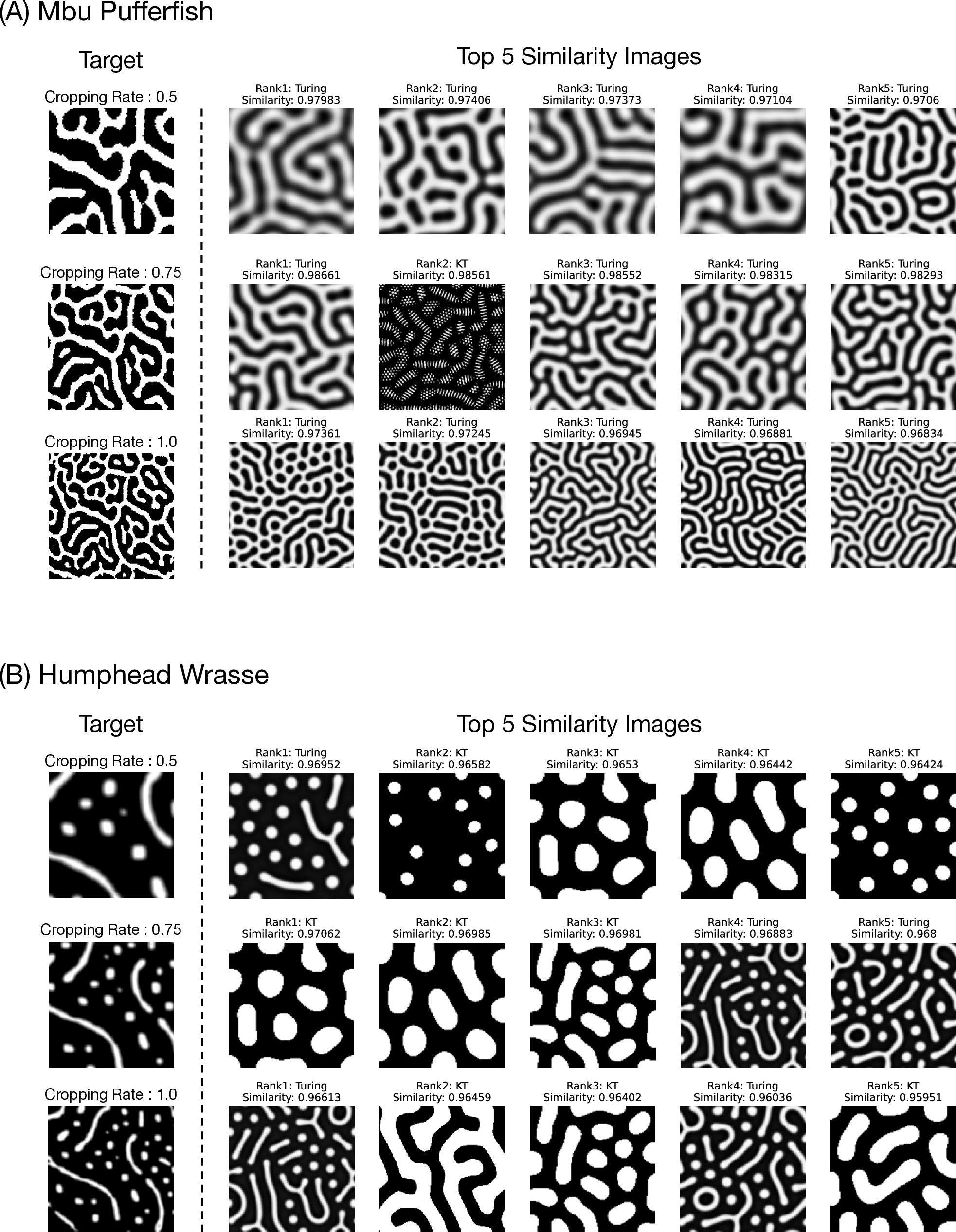

Supplement: S14 Fig — The relationship between the image cropping applied as a preprocessing step in model selection and the top five most similar pattern images is illustrated using skin pattern of the humphead wrasse (Cheilinus undulatus) as an example. (TIF) [file pcbi.1012689.s019.tif]

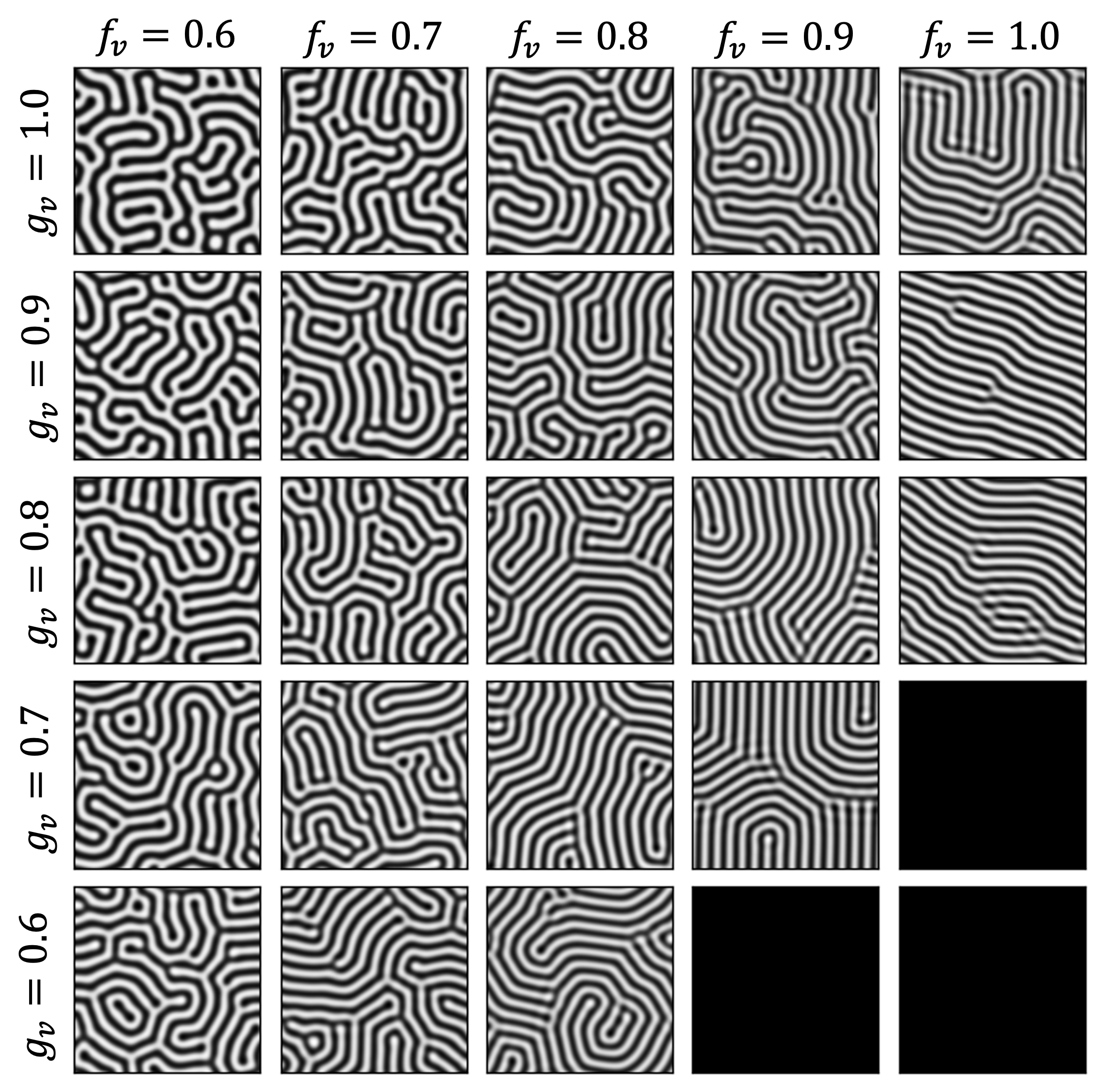

Supplement: S15 Fig — Each pattern image is one example generated by Turing model with the corresponding parameters. The horizontal axis represents fv, and the vertical axis represents gv. Patterns corresponding to the parameters in the bottom right area of the figure cannot be generated. (TIF) [file pcbi.1012689.s020.tif]

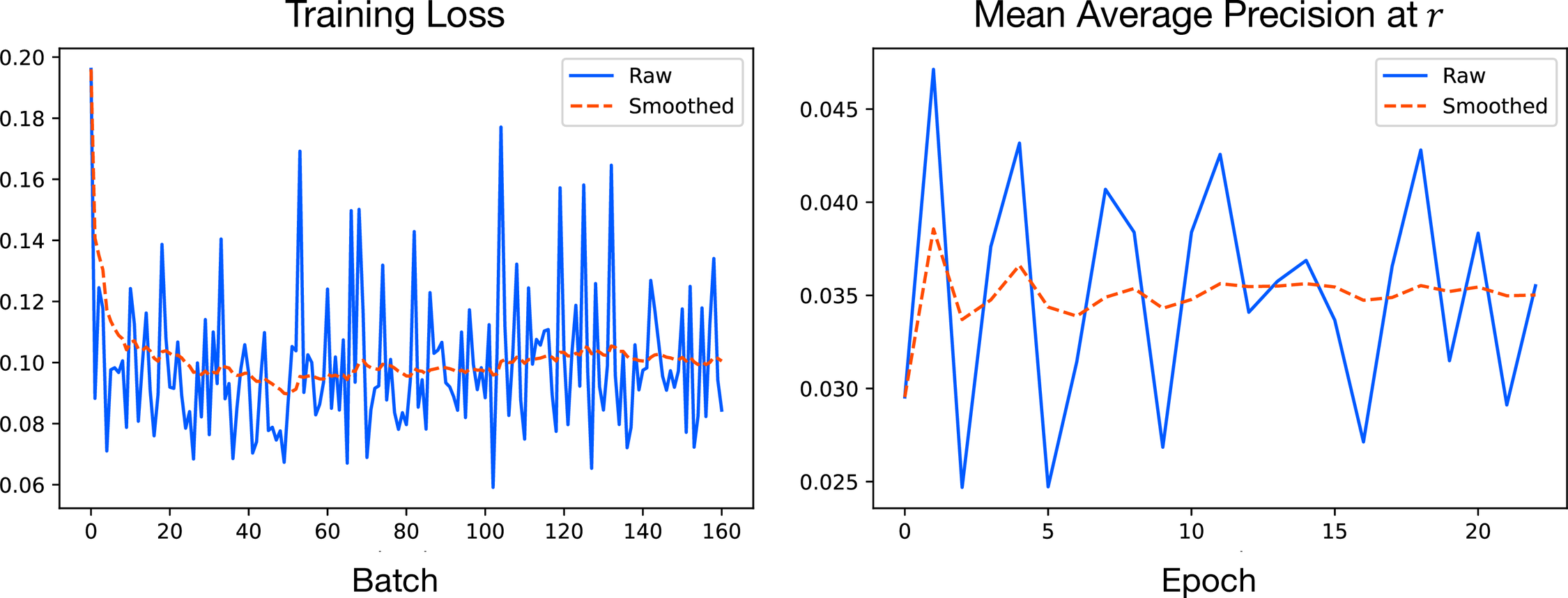

Supplement: S16 Fig — (A) Training loss was calculated for each batch. The blue line represents the raw values, while the red dashed line indicates the smoothed values. (B) MAP@k was calculated at the end of each epoch. The blue line represents the raw values, and the red dashed line indicates the smoothed values. The smoothing process was consistent with the method used in TensorBoard. A weighted average was calculated between the previous value and the current value in a 0.95: 0.05 ratio, and the result was divided by 1 − 0.95t as a debiasing step, where t represents the number of data points up to the previous point. (TIF) [file pcbi.1012689.s021.tif]

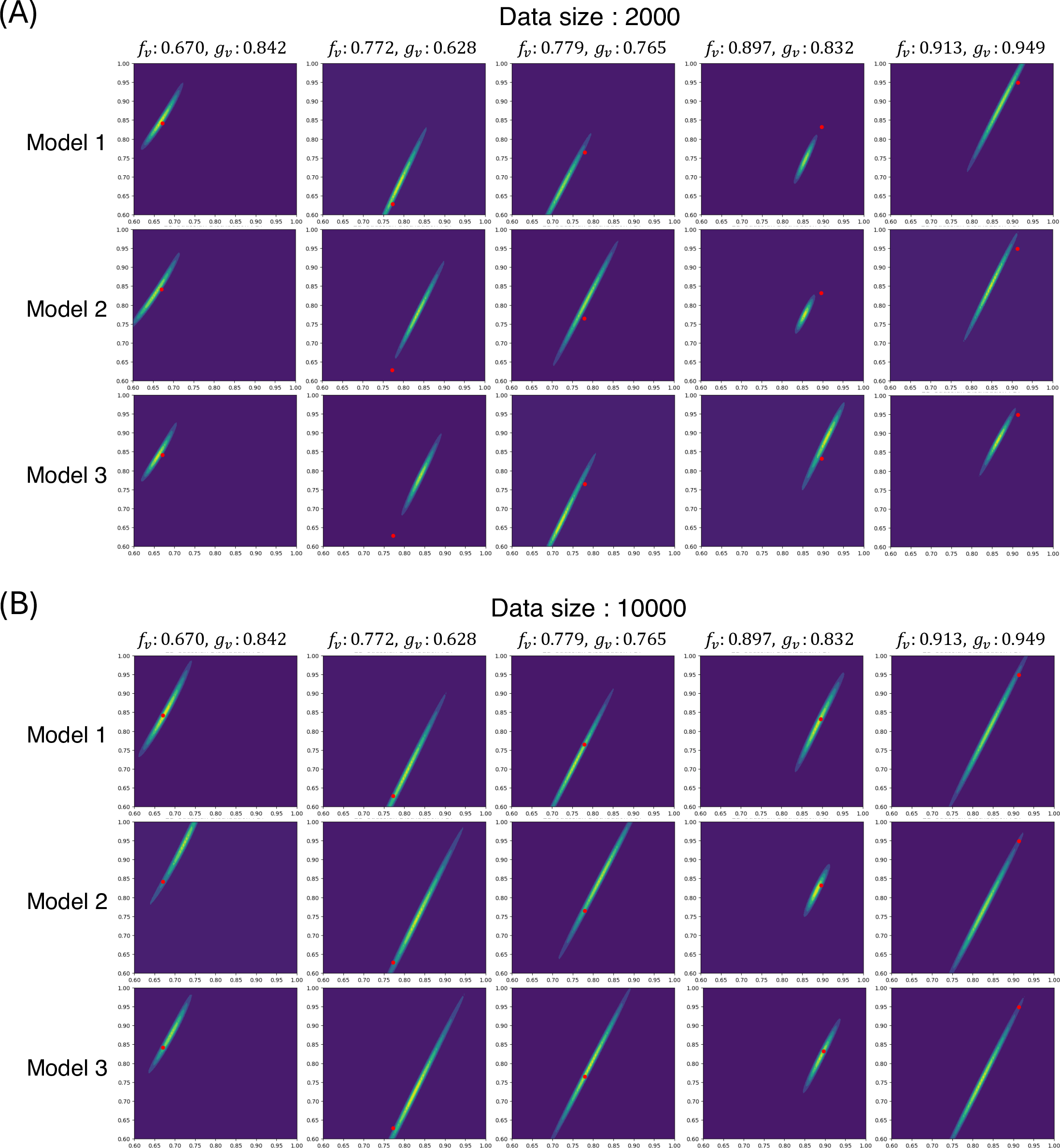

Supplement: S17 Fig — SD-NPE was initialized with different random seeds to estimate the parameters fv and gv of the Turing model. The predictions are shown for five different parameter sets used as test cases for each model. The data size of training dataset was 2000 (A) and 10000 (B). (TIF) [file pcbi.1012689.s022.tif]
